# Efficient viral delivery of Cas9 into human safe harbor

**DOI:** 10.1038/s41598-020-78450-8

**Published:** 2020-12-08

**Authors:** Hideki Hayashi, Yoshinao Kubo, Mai Izumida, Toshifumi Matsuyama

**Affiliations:** 1 Medical University Research Administrator, Nagasaki University School of Medicine, Nagasaki, Japan; 2 Department of Clinical Medicine, Institute of Tropical Medicine, Nagasaki University, Nagasaki, Japan; 3 Department of Pathology, Graduate School of Biomedical Sciences, Nagasaki University, Nagasaki, Japan

**Keywords:** Biological techniques, Genetics

## Abstract

Gene editing using CRISPR/Cas9 is a promising method to cure many human genetic diseases. We have developed an efficient system to deliver Cas9 into the adeno-associated virus integration site 1 (AAVS1) locus, known as a safe harbor, using lentivirus and AAV viral vectors, as a step toward future in vivo transduction. First, we introduced Cas9v1 (derived from *Streptococcus pyogenes*) at random into the genome using a lentiviral vector. Cas9v1 activity was used when the N-terminal 1.9 kb, and C-terminal 2.3 kb fragments of another Cas9v2 (human codon-optimized) were employed sequentially with specific single-guide RNAs (sgRNAs) and homology donors carried by AAV vectors into the AAVS1 locus. Then, Cas9v1 was removed from the genome by another AAV vector containing sgRNA targeting the long terminal repeat of the lentivirus vector. The reconstituted Cas9v2 in the AAVS1 locus was functional and gene editing was efficient.

## Introduction

The clustered regularly interspaced short palindromic repeat (CRISPR)/Cas9 system is widely used for genome editing^1,2^. Introduction of inducible Cas9 into the adeno-associated virus integration site 1 (AAVS1) locus in human stem cell lines, ex vivo*,* provides a platform for subsequent gene editing^3,4^. The AAVS1 locus, in intron 1 of PPP1R12C (protein phosphatase 1 regulatory subunit 12C), is known as a “safe harbor” because its disruption does not have adverse effects on the cell, and robust transcription can be used to maintain the expression of an exogenously inserted gene^5–7^. For precise gene editing using homology-directed repair (HDR), the Cas9 gene, single-guide RNA (sgRNA), and homology arms must be introduced. Cas9 delivery is usually performed using transfection or electroporation, in vitro*,* as it is too large to pack into an adeno-associated virus (AAV) vector bundled with homology arms^8,9^. To solve this problem, SaCas9 from *Staphylococcus aureus*, which is smaller than SpCas9, was used to package the AAV vector^10^. Previous studies have reported the expression of Cas9 N- and C-terminal segments in different vectors, followed by reconstitution using either intein-mediated trans-splicing or a photo-inducible dimerization system^11,12^. Non-integrating lentiviral or adenovirus vectors have also been used for Cas9 delivery^13,14^. Here, we propose a new method, using only viral vectors, to efficiently integrate Cas9 into the AAVS1 locus by exploiting the advantages of lentiviral and AAV vectors for future in vivo applications. Both specific sgRNA and homologous arms are small enough to be packed into an AAV vector, which is considered the safest viral vector for use in vivo^15^. If Cas9 is integrated into the AAVS1 locus of target cells, its stable expression will efficiently enable precise gene editing. The AAVS1 site-integrated Cas9 was also used for the effective removal of the lentivirus containing Cas9 integrated into the genome at random^16,17^. Given the capacity of the AAV vector between inverted terminal repeats (ITRs) of ~ 4.7 kb, the actual size of the introducing gene is limited to approximately 2.5 kb. For precise gene editing using HDR, the vector needs to carry an additional 1.9–2.2 kb [0.4 kb for sgRNA with U6 promoter, 0.6 kb for left and right homology arms, 0.7 kb or 0.4 kb for puromycin or blasticidin resistance gene with a 0.3 kb polyadenylation signal, and small connecting fragments such as targeting sites, splice acceptor, T2A (*Thosea asigna* virus 2A), and P2A (porcine teschovirus-1 2A) self-cleaving peptides]. Therefore, we have to introduce the Cas9 N- and C-terminal halves sequentially. Here, we present a basic four-step strategy to efficiently introduce Cas9 into the AAVS1 locus, followed by a modified protocol to reduce off-target effects. The modified protocol could be further improved by employing both the modified Cas9s to minimize off-target effects and using non-integrating lentivirus to reduce the genetic risk^13,18^.

## Results

### Integration of wild-type Cas9 into the AAVS1 locus

**Figure 1:**
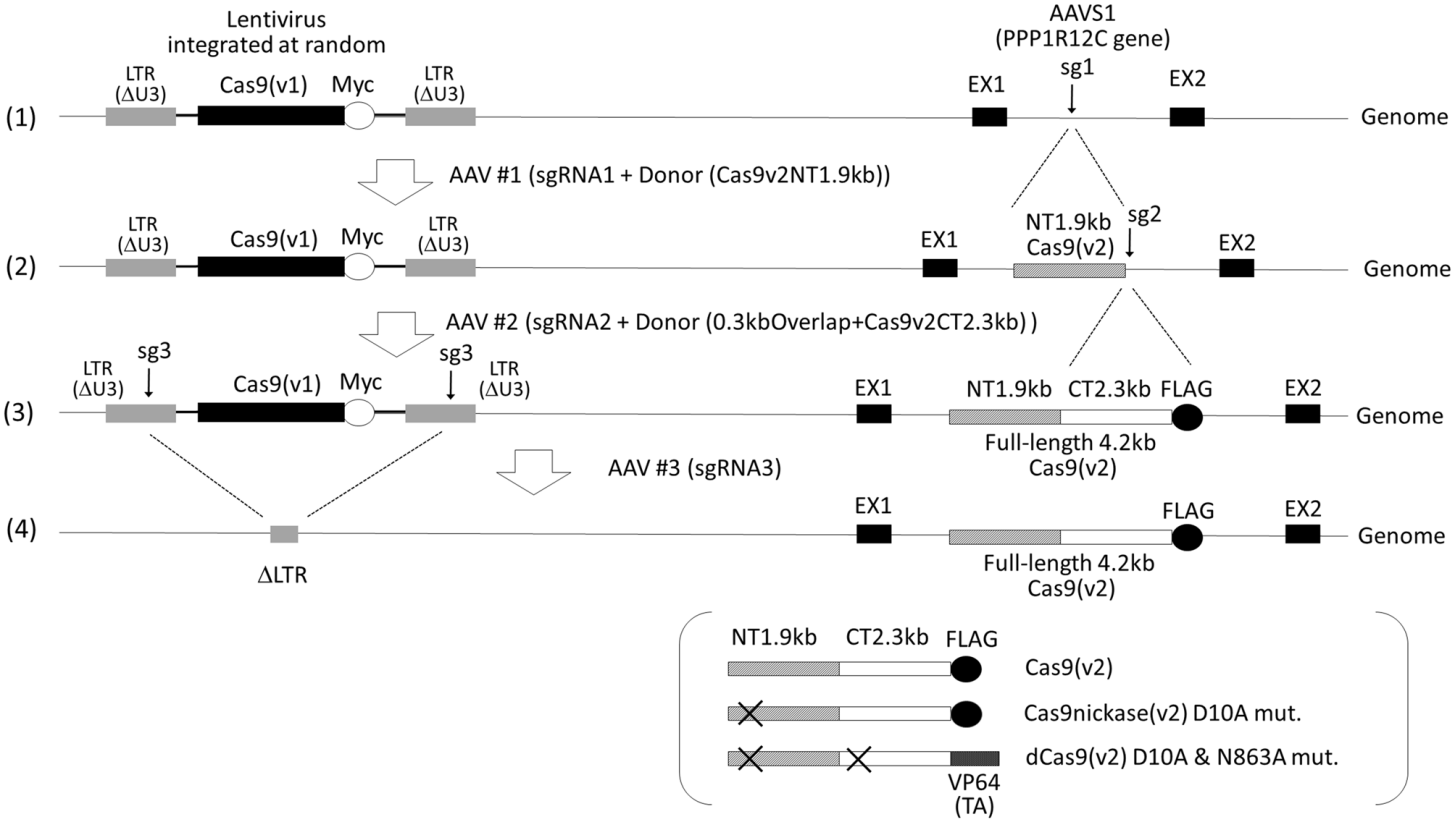
Schematic diagram of the four-step integration strategy of Cas9 into the AAVS1 locus. (1) The first step is to generate a cell line expressing Cas9v1 by random integration of the recombinant lentivirus in the host genome. (2) Using the Cas9v1 nuclease activity, the N-terminal 1.9 kb fragment of Cas9v2 was introduced into the AAVS1 locus by AAV vector #1 with the sgRNA1-targeting AAVS1 site and the homology arms flanking the Cas9 N-terminal 1.9 kb fragment. (3) full-length Cas9v2 was reconstituted in the AAVS1 locus using HDR, with AAV vector #2 providing the 2.3 kb Cas9v2 C-terminal fragment, as well as a 0.3 kb region overlapping the 1.9 kb N-terminal fragment; (4) lentivirus, containing Cas9v1, was removed from the genome using AAV vector #3, which contained sgRNA targeting long terminal repeats (LTR) in the lentiviral vector. The schematic structures of wild-type Cas9v2, Cas9v2 nickase (Cas9nikase), and dead Cas9v2 (dCas9) made by the same procedures as wild-type Cas9v2 are shown in brackets. The sgRNAs targeting AAVS1, the 3′-end of the Cas9v1 N-terminal 1.9 kb fragment and the LTR of the lentivirus are shown as sg1, sg2, and sg3, respectively. AAVS1, adeno-associated virus integration site 1 (intron1 of the PPP1R12C, protein phosphatase 1 regulatory subunit 12C); EX, exon; Cas9v1, S. pyogenes-derived Cas9; Cas9v2, human codon-optimized Cas9; VP64, Herpes simplex virus transcriptional activator domain (TA).

We developed a method to integrate Cas9 into the AAVS1 locus using only viral vectors; thus, exploiting the advantages of both lentiviral and AAV vectors. Stably expressed Cas9 enables precise gene editing after transduction with AAV vectors containing specific sgRNAs and homologous arms, thus enabling HDR^15^. As shown in Fig. 1, we used two types of Cas9: Cas9v1, original SpCas9 (*Streptococcus pyogenes*), and Cas9v2, human codon-optimized from SpCas9^19^. Although they encode for the same protein, Cas9 differs in its DNA sequence, enabling sgRNA2 in AAV vector #2 to recognize Cas9v2, but not Cas9v1. To monitor the expression of both Cas9s at the protein level, we added Myc-tag and FLAG-tag to Cas9v1 and Cas9v2, respectively (Fig. 1). In addition, we employed a double-cut donor method, with 0.3 kb short homology arms, to enable HDR and minimize the size of insertion DNA for AAV vectors^20,21^. The procedure comprised the following four steps: (1) Cas9v1 (*S. pyogenes derived*) was randomly introduced into the genome of host cells using a lentiviral vector; this could accommodate the large size of Cas9v1, and ensure its robust and stable expression, though the integration site was uncontrolled; (2) Cas9v1 nuclease activity was used to introduce the 1.9 kb Cas9v2 N-terminal fragment (human codon-optimized) into the AAVS1 locus via the AAV vector #1, as its exogenous gene capacity was ~ 2.5 kb (~ 2.2 kb is used for sgRNA, homology arms, connecting peptides, a puromycin resistant gene and polyadenylation signal); (3) full-length Cas9v2 was reconstituted in the AAVS1 locus using HDR, with the AAV vector #2 providing the 2.3 kb Cas9v2 C-terminal fragment, as well as a 0.3 kb region overlapping the 1.9 kb N-terminal fragment; (4) lentivirus, containing Cas9v1, was removed from the genome using the AAV vector #3, which contained sgRNA targeting long terminal repeats (LTR) in the lentiviral vector.

1. Random integration of Cas9v1-Myc into the genome

First, we introduced Cas9v1-Myc into the host genome of human embryonic kidney (HEK) 293 T cells by random integration using a lentiviral vector. For robust and ubiquitous expression in many cells, both the Cas9v1-Myc transcript and the hygromycin resistance gene, connected with a T2A self-cleaving peptide, were driven via a cytomegalovirus (CMV) promoter. Transduced HEK293T cells were selected with hygromycin B for two weeks. All five hygromycin-resistant clones (100%) expressed Cas9v1-Myc (Supplementary Fig S1).

(2) Integration of the 1.9 kb Cas9v2 N-terminal fragment into the AAVS1 locus

**Figure 2:**
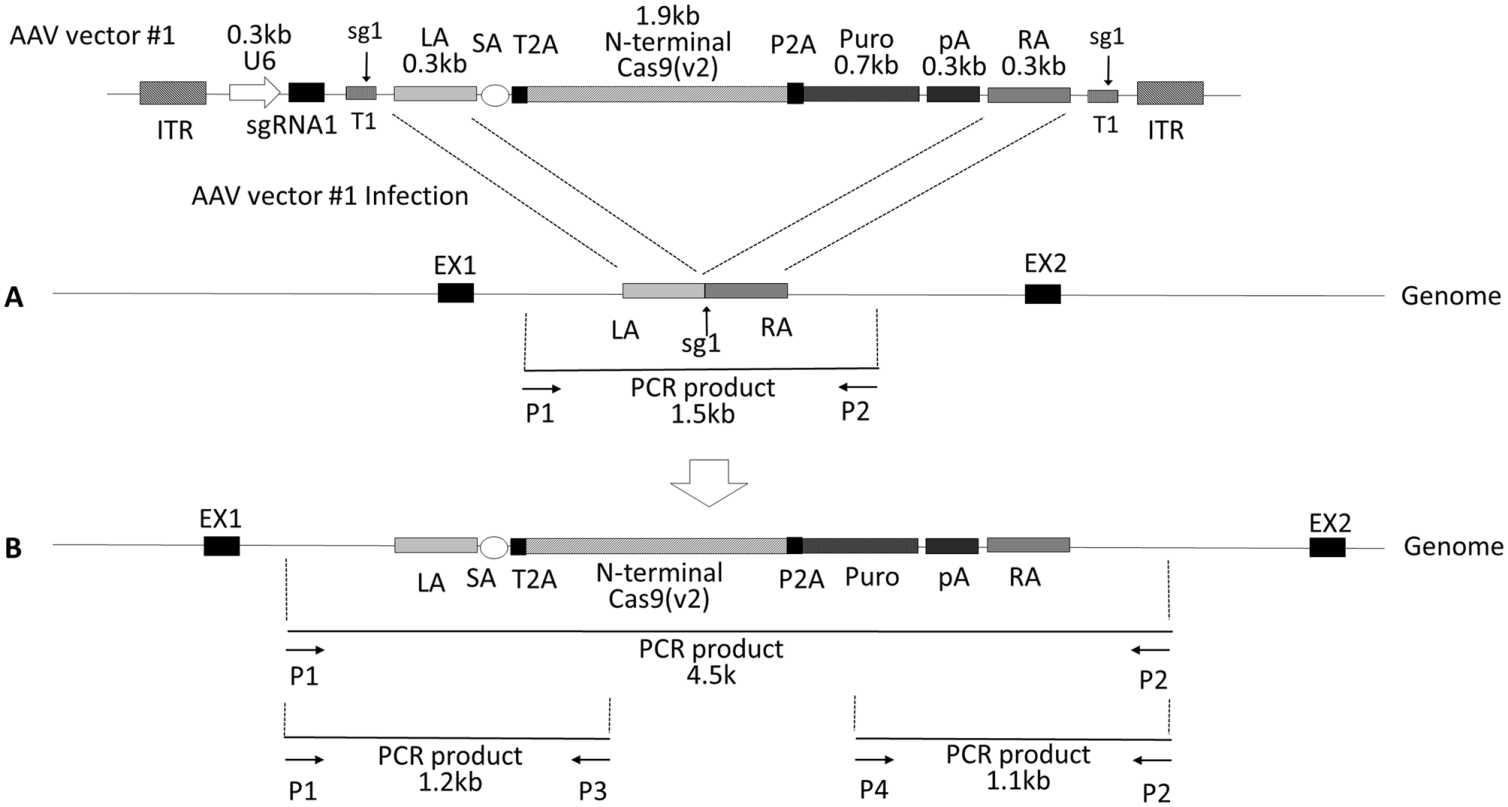
AAV vector #1 construct for introduction of N-terminal 1.9 kb Cas9v2 into the AAVS1 locus. (**A**) AAV vector #1 is designed to introduce the N-terminal Cas9v2 into the AAVS1 locus as a double-cut donor. The vector sequence is shown in Supplementary Table S1. T1 is the AAVS1 target site including the protospacer adjacent motif (PAM). The T1 sequence was synthesized and added adjacent to each homology arm to cut out the donor DNA from the AAV vector genome. U6, U6 RNA polymerase III promoter; sg1, AAVS1-specific sgRNA1; LA and RA, left and right homology arms; SA, splicing acceptor; T2A and P2A: *Thosea asigna* virus and porcine teschovirus-1 2A self-cleaving peptides; Puro, puromycin resistance gene; pA, a polyadenylation signal sequence; ITR, inverted terminal repeat. (**B**) Genome structure after integration of the donor by HDR. Primers P1–P2 for PCR were placed outside of the homology arms. Primers P1–P3 and P4–P2 were used to detect the N-terminal Cas9 and the Puro genes, respectively. The PCR primer sequences are listed in Supplementary Table S3.

**Figure 3:**
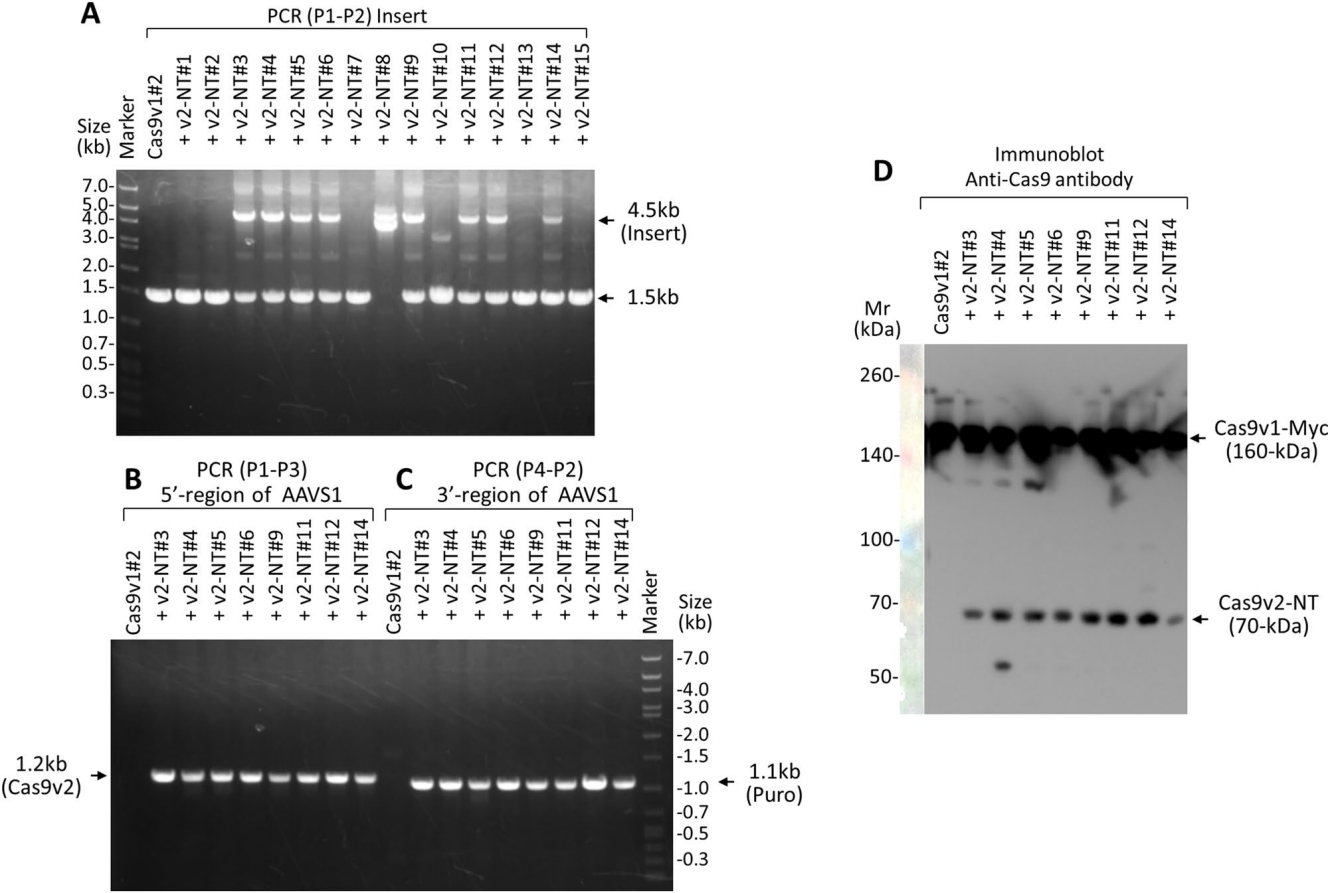
PCR and immunoblot to confirm N-terminal Cas9v2 genomic integration and expression. (**A**) Genomic DNA from parent HEK293T-Cas9v1-Myc cells (Cas9v1#2) and the 293 T-Cas9v1-Myc cells expressing the 1.9 kb N-terminal Cas9v2 after AAV vector #1 infection and puromycin selection (+ v2-NT #1–#15) was analyzed by PCR with primers P1–P2 (4-min extension at 68 °C). The 4.5 kb and 1.5 kb PCR products are from the N-terminal Cas9v2-integrated and non-integrated original alleles, respectively. The Cas9v2 NT #8 clone had 4.5 kb and 4.2 kb PCR products. (**B**) Further analysis of the Cas9v2-integrated clones with primers P1–P3 (1.2 kb for Cas9v2). (**C**) Further analysis of the Cas9v2-integrated clones with primers P4–P2 (1.1 kb for Puro). **D.** The lysates obtained from the cells used for (**B**,**C**) were analyzed by immunoblot using an antibody against Cas9 that recognizes both Cas9s, after 6% SDS-PAGE. The arrows indicate the Cas9v1-Myc (160 kDa) and the 1.9 kb N-terminal Cas9v2 (70 kDa).

Using Cas9v1-Myc nuclease activity, a 1.9 kb Cas9v2 N-terminal fragment was introduced into the AAVS1 locus using an AAV vector (Fig. 2). To enable HDR, AAV vector #1 was designed to express AAVS1-specific sgRNA1 (sg1), and to provide both a double-cut donor, with 0.3 kb short homology arms, and T1 targeting sequences, including a protospacer adjacent motif (PAM)^20,21^. The complete sequence of AAV #1 is shown in Supplementary Table S1. When HDR occurs, both the 1.9 kb Cas9v2 N-terminal and the puromycin resistance gene are expressed from the robust PPP1R12C transcript, via a splice acceptor (SA), T2A, and P2A self-cleaving peptides. HEK293T cells expressing Cas9v1-Myc (clone #2) were transduced with the AAV vector #1 and then selected for 2 weeks using puromycin. Genomic DNA, prepared from puromycin-resistant cells (clones #1–#15), was analyzed using PCR, with primers P1–P2 set outside the homology arms (Fig. 3A). Primer sequences are listed in Supplementary Table S3. The 4.5 kb and 1.5 kb PCR products were from the N-terminal of integrated Cas9v2 and nonintegrated original alleles, respectively. Eight of the 15 clones examined (53%) had two bands, indicating that only one allele had N-terminal Cas9v2, while only the clone #8 had inserts (4.5 kb and 4.2 kb) in both alleles. Possible Cas9v2-integrated clones (PCR 4.5 kb product) were further analyzed using primers P1–P3 and P4–P2. All eight candidates had the Cas9v2 gene (Fig. 3B: 1.2 kb using primers P1–P3) and puromycin (Fig. 3C: 1.1 kb using primers P4–P2). In addition, following 6% SDS-PAGE, lysates obtained from the cells used for PCR analysis were analyzed using immunoblot with an antibody recognizing both Cas9s. All eight clones expressed the 1.9 kb N-terminal of Cas9v2 (70 kDa) as well as Cas9v1-Myc (160 kDa) (Fig. 3D). The 4.5 kb sequence of the N-terminal (NT) clone #4 fragment was determined by Sanger sequencing and confirmed the gene structure outlined in Fig. 2B. The DNA sequence of clone #4 is listed in Table S2. The 4.5 kb fragment from clone #8 was the same as that of clone #4; however, the 4.2 kb fragment from clone #8 possessed both an insertion and deletion around the right homology arm (Supplementary Fig S2).

(3) Reconstitution of full-length Cas9v2-FLAG at the AAVS1 locus

**Figure 4:**
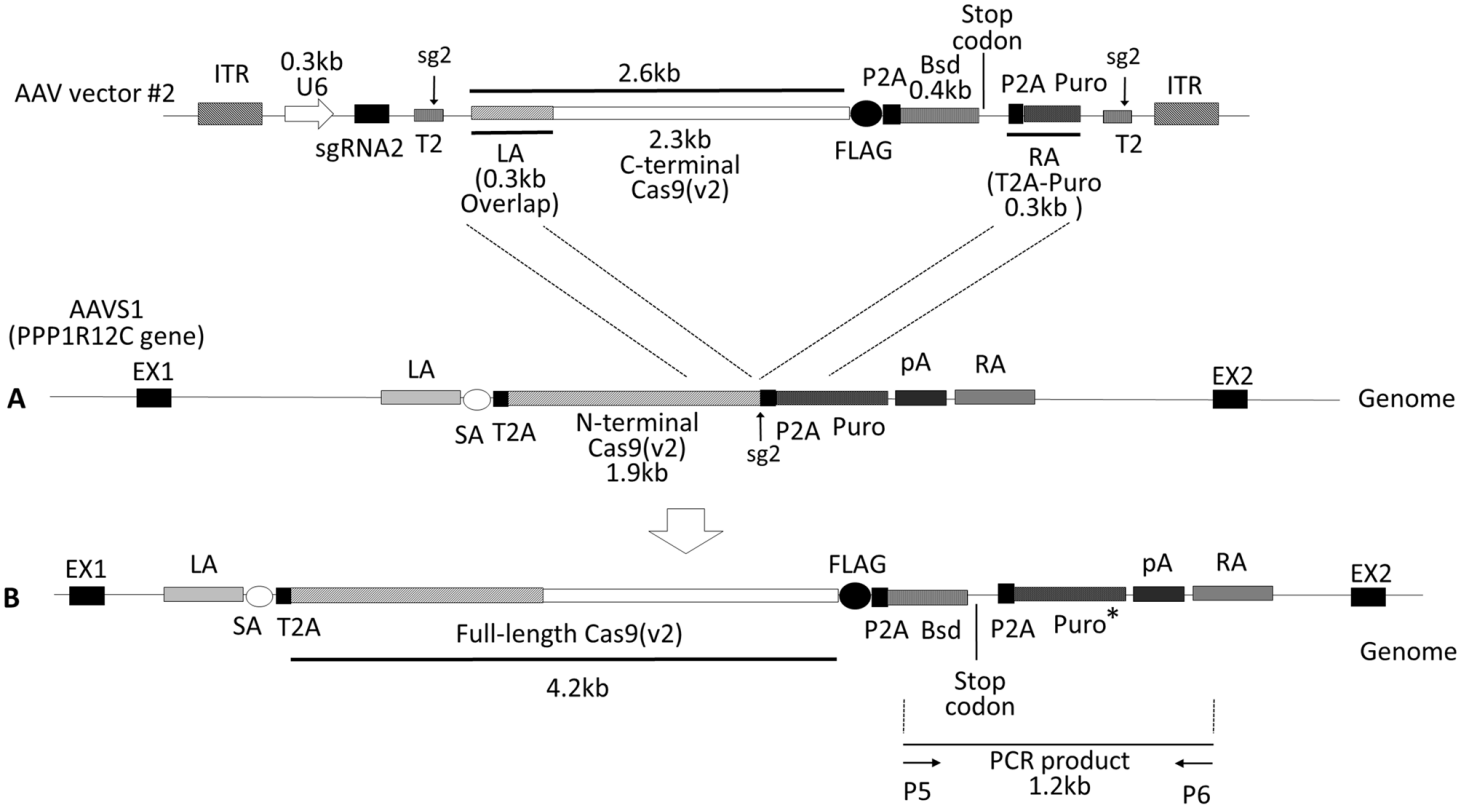
AAV vector #2 construct used to reconstitute full-length Cas9v2-FLAG in the AAVS1 locus. (**A**) AAV vector #2 was designed to reconstitute full-length Cas9v2 as a double cut donor. T2 is the target site of the junction between the 3′-end of 1.9 kb N-terminal Cas9v2 and the 5′-end of P2A (a self-cleaving peptide) with PAM. The 2.6 kb C-terminal fragment has a 0.3 kb region overlapping with the 1.9 kb N-terminal Cas9v2 fragment to reconstitute full-length Cas9v2 by HDR. The T2 sequence was added adjacent to each homology arm to cut out the donor DNA from the AAV vector genome. U6, U6 promoter; sg2, the target site T2-specific sgRNA2; Bsd, blasticidin resistance gene. (**B**) Genome structure after integration of the donor by HDR. Primers P5–P6 were used to detect the Bsd gene in the AAVS1 locus after HDR. The sequences of AAV vector #2 and PCR primers are shown in Supplementary Tables S1 and S3, respectively. *The puromycin-resistant gene is not translated to the protein because of the stop codon following the blasticidin resistance gene.

**Figure 5:**
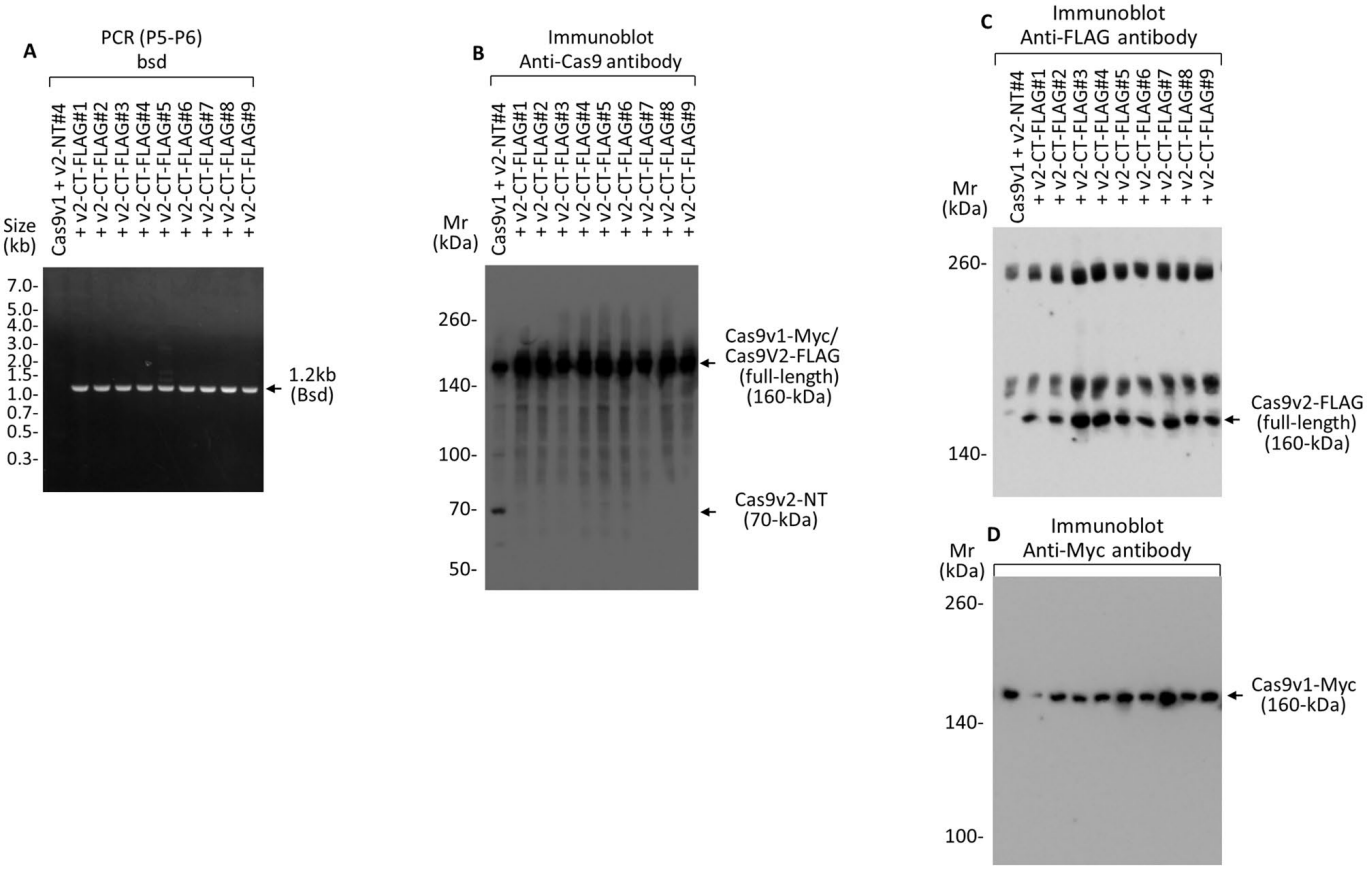
PCR and immunoblot to confirm the reconstitution of full-length Cas9v2-FLAG in AAVS1 locus. (**A**) PCR analysis of genomic DNA prepared from the HEK293T-Cas9v1-Myc + Cas9v2-NT #4 cells and the HEK293T-Cas9v1-Myc cells expressing full-length Cas9v2-FLAG (+ v2-CT-FLAG #1–#9), after AAV vector #2 infection and blasticidin selection. The DNA was amplified by primers P5–P6 and the 1.2 kb PCR products indicating the integration of donor elements (Bsd gene) were detected in all clones. (**B**–**D**) Lysates obtained from the cells used in (**A**) were analyzed by immunoblot with an antibody against Cas9 that recognizes both Cas9s, after 6% SDS-PAGE. The 70-kDa band indicates the 1.9 kb N-terminal Cas9v2 (**B**). The 160-kDa band was confirmed with the specific tag antibodies to contain the reconstituted full-length Cas9v2-FLAG (**C**) and Cas9v1-Myc (**D**).

To reconstitute full-length Cas9v2-FLAG using HDR, we introduced a 2.6 kb fragment containing the 2.3 kb C-terminal (CT) fragment of Cas9v2-FLAG and the 0.3 kb region overlapping the 1.9 kb N-terminal fragment, via the AAV vector #2 (Fig. 4). sgRNA2 (sg2) targets the junction sequence (T2) between the 3′-end of the 1.9 kb N-terminal Cas9v2 and the 5′-end of the P2A self-cleaving peptide. P2A, and the 0.3 kb N-terminal fragment of puromycin, were used as the right homology arm; to provide a double-cut donor, the fragment flanked the T2 target sequence and ITR (Supplementary Table S1). P2A expresses a blasticidin resistance gene as well as reconstituted full-length Cas9v2-FLAG, from the PPP1R12C promoter-driven transcript present at the AAVS1 site. The puromycin-resistant gene was also reconstituted using HDR; however, it was not translated to protein due to the stop codon following the blasticidin resistance gene. The AAV vector #2 was introduced into HEK293T cells expressing both Cas9v1-Myc and the 1.9 kb N-terminal of Cas9v2 (NT clone #4); they were then selected with blasticidin for 2 weeks. Blasticidin-resistant cells (clones #1–#9) were sensitive to puromycin, and their genomic DNA was prepared and analyzed using PCR, with primers P5–P6 (Fig. 5A). Donor element integration was indicated by 1.2 kb PCR products detected in all nine clones. Following 6% SDS-PAGE, lysates from the same cells were analyzed by immunoblotting with an antibody recognizing both Cas9s (Fig. 5B). In all the AAV vector #2-transduced cells, the 70 kDa band, indicating the 1.9 kb N-terminal of Cas9v2, disappeared, while 160 kDa bands increased in intensity. These bands were confirmed to have reconstituted full-length Cas9v2-FLAG and Cas9v1-Myc with anti-FLAG (Fig. 5C) and anti-Myc antibodies (Fig. 5D), respectively. The sequence of Cas9v2 full-length clone #7, including the AAVS1 integration site, was confirmed via Sanger sequencing (Supplementary Table S2).

(4) Removal of randomly integrated lentivirus, containing Cas9v1-Myc, from the genome

**Figure 6:**
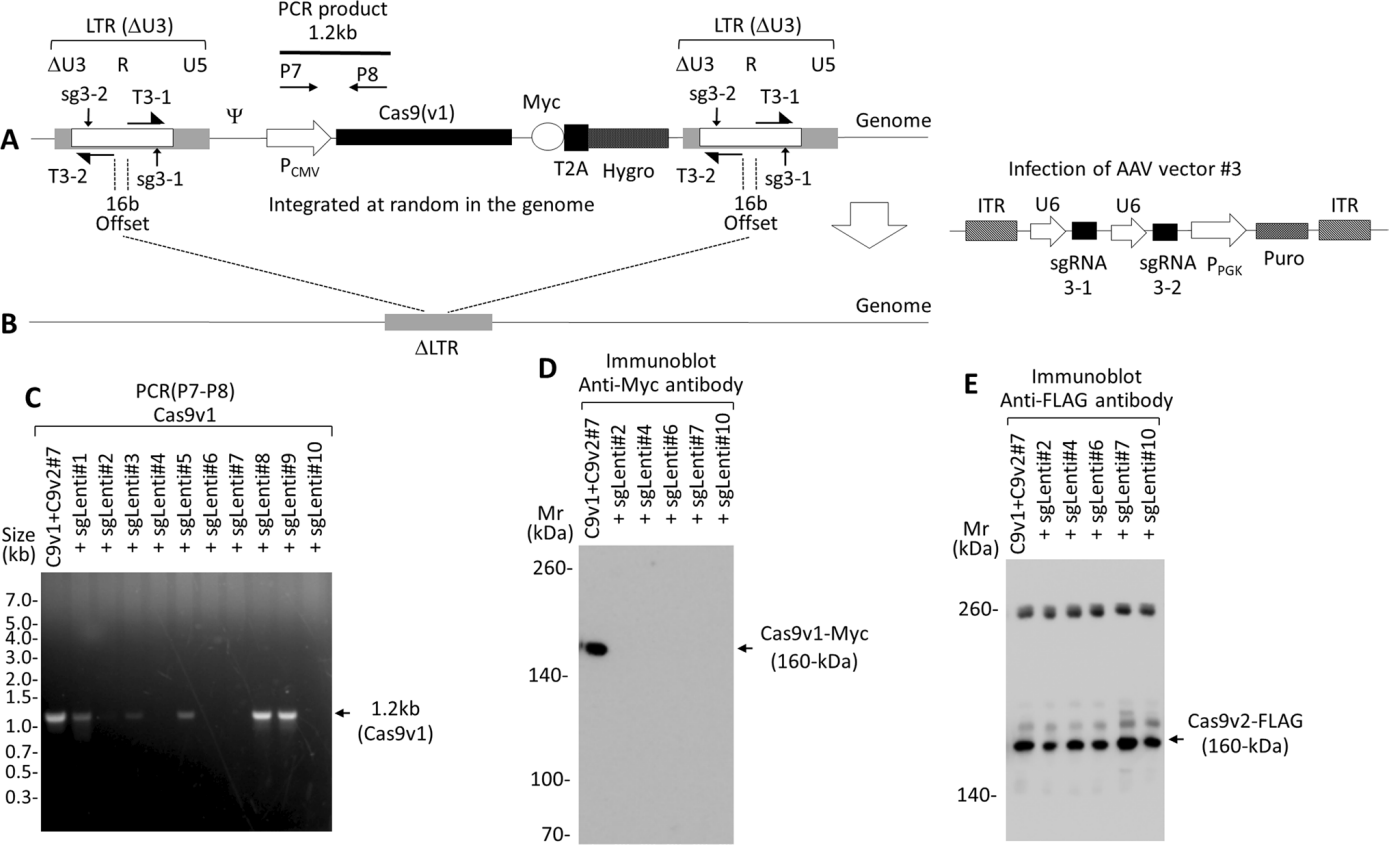
AAV vector #3 construct targeting the genome integrated lentivirus long terminal repeat (LTR). (**A**,**B**) AAV vector #3 targeting the LTR of the lentiviral vector (T3) was created by placing a pair of sgRNAs (sg3-1 and sg3-2) with 16 bp offset for DSB (double-strand break) by Cas9 nickase as well as wild-type Cas9 and placing a puromycin resistance gene between the ITRs. Primers P7–P8 for Cas9v1 are used to examine whether the lentivirus containing Cas9v1-Myc is removed by NHEJ with AAV vector #3 infection (**B**) The sgRNA and PCR primer sequences are shown in Supplementary Tables S1 and S3, respectively. (**C**–**E**) PCR analysis of genomic DNA prepared from the HEK293T-Cas9v1-Myc + full-length Cas9v2-FLAG #7 cells, and the HEK293T-Cas9v1-Myc + Cas9v2-FLAG cells (sgLenti #1–#10) expressing sgRNA3 targeting the lentivirus LTR, after AAV #3 vector infection and puromycin selection. The DNA was amplified using primers P7–P8 (for Cas9v1) (**C**). The lysates from the cells that had no 1.2 kb PCR products in (**C**) were analyzed by immunoblot for Cas9v1-Myc against Myc-tag (**D**) and Cas9v2-FLAG with an antibody against FLAG-tag (**E**).

To remove randomly integrated lentivirus, containing Cas9v1-Myc, from the genome, we constructed the AAV vector #3, which used non-homologous end joining (NHEJ) to target the LTR of the lentiviral vector (T3) (Fig. 6A,B). To select cells expressing sgRNA, we used a puromycin resistance gene, as cells expressing Cas9v1-Myc and full-length Cas9v2-FLAG lost functional puromycin expression (Fig. 4B). Previous reports indicate that sgRNA targeting LTR of HIV successfully removed genes between the LTR in many cases^16,17^. We set a pair of sgRNAs (sg3-1 and sg3-2), with a 16 bp offset, for a DNA double-strand break (DSB) using Cas9 nickase as well as wild-type Cas9 (Fig. 6A). Nickase requires a pair of offset (usually − 4 to 20 bp) sgRNAs, complementary to the opposite strand of the target site^22,23^. HEK293T cells expressing both Cas9v1-Myc and full-length Cas9v2-FLAG (Cas9v2 full-length clone #7) were transduced with the AAV vector #3 and then selected with puromycin. Puromycin-resistant cells (clones #1–#10) were analyzed using PCR with specific primers (P7–P8) for Cas9v1-Myc (Fig. 6C) and immunoblotting for Cas9v1-Myc (Fig. 6D)and Cas9v2-FLAG (Fig. 6E). Lentivirus containing Cas9v1-Myc was successfully removed from the genome in five out of ten (50%) puromycin-resistant colonies by NHEJ using the AAV vector #3 (MOI = 0.1), leaving only small pieces of the LTR. Although the removal rate among puromycin-resistant cells was not unpropitious, the overall removal rate including puromycin-sensitive cells was about 1%. Specific removal of Cas9v1, compared to Cas9v2, was confirmed using PCR and immunoblot analyses.

(5) Verification of reconstituted Cas9v2-FLAG function

To verify that the reconstituted Cas9v2-FLAG at the AAVS1 locus was functional for nuclease activity, we examined whether it could produce DSB at a specific site, with sgRNA targeting the site. We chose the interferon alpha and beta-receptor subunit 1 (IFNAR1) gene as target^24,25^. The AAV vector #4, targeting exon 1 of IFNAR1 (T4), was created by placing a pair of sgRNAs (sg4-1 and sg4-2) with a 7 bp offset, for DSB using Cas9 nickase as well as wild-type Cas9 (Supplementary Fig S3). The entire sequence of the AAV vector #4 is shown in Supplementary Table S1. To select cells containing sgRNA, the hygromycin resistance gene was placed between ITRs, as this gene was removed with Cas9v1-Myc in cells expressing the sgRNA3 targeting lentivirus LTR (Fig. 6A,B). Cells (Cas9v2-FLAG + sgLenti#10) were then infected with the AAV vector #4, and six hygromycin-resistant clones (#1–#6), expressing Cas9v2-FLAG and sgIFNAR1, were obtained. We analyzed the genomic structure of IFNAR1 in clones #1 and #3 (Supplementary Fig S4). The cells had heterogeneous insertions and deletions in IFNAR1 alleles. Therefore, we confirmed that reconstituted Cas9v2-FLAG is valid for use in editing other genes.

### Introduction of Cas9 nickase in the AAVS1 locus

Although reconstituted Cas9v2-FLAG in the AAVS1 locus was functional, Cas9 may sometimes cause adverse off-target effects. To reduce the possibility of off-target effects in gene editing, we also introduced Cas9v2 nickase with a D10A mutation into the AAVS1 locus^22,23^. To transduce HEK293T cells expressing Cas9v1-Myc (clone #2), we used the 1.7 kb N-terminal of Cas9v2, with a D10A mutation (AAV vector #5 in Supplementary Fig S5), instead of the AAV vector #1. Three out of ten puromycin-resistant clones (#1–#10) (30%) possessed the N-terminal Cas9v2 nickase, and the immunoblot analyses of independent two clones (Cas9v1-Myc #2 + Cas9v2nickase-NT#7 and #9) are shown in Supplementary Fig S6.

To reconstitute full-length Cas9v2-FLAG nickase, we used the same AAV vector #2 as in Fig. 4 to transduce cells expressing both Cas9v1 and the mutated 1.9 kb N-terminal of Cas9v2 (nickase-NT clone #9) (Supplementary Fig S7). All 10 cell lines resistant to blasticidin (#1–#10) (100%) expressed both Cas9v1-Myc and full-length Cas9v2nickase-FLAG. Immunoblot analyses of independent two clones (Cas9v1-Myc #2 + Cas9v2nickase (full)-FLAG#3 and #4) are shown in Supplementary Fig S6. The sequence of full-length Cas9v2nickase-FLAG #3, including the AAVS1 integration site, was confirmed via Sanger sequencing (Supplementary Table S2).

Finally, we removed the lentiviral vector, containing Cas9v1-Myc, from the genome (full-length Cas9v2nickase-FLAG #3) using the same AAV vector #3 as in Fig. 6. Lentivirus containing Cas9v1-Myc was removed from the genome in 3 out of 10 clones (30%), and immunoblot analyses of two clones (Cas9v2nickase (full)-FLAG#3 + sgLenti#4 and #5) are shown in Supplementary Fig S6.

### Introduction of dead Cas9 (dCas9) into the AAVS1 locus

The CRISPR/Cas9 system is used not only for the knockout or knock-in of specific genes but also for activation of specific gene transcription^26^. We reconstituted full-length nuclease-free dead Cas9, carrying D10A and N876A mutations, and conjugated to VP64 Herpes simplex virus transcriptional activator domain (dCas9v2-VP64) in the AAVS1 locus by transducing HEK293T cells expressing both Cas9v1 and D10A-mutated Cas9v2 nickase-NT#9 with the AAV vector #6 (Supplementary Fig S8). All 10 blasticidin-resistant cell lines (100%) expressed the reconstituted full-length dCas9v2-VP64 as well as Cas9v1-Myc. Immunoblot analyses of two independent clones (Cas9v1-Myc #2 + dCas9v2 (full)-VP64 #1 and #2) are shown in Supplementary Fig S6. The sequence of full-length dCas9v2-VP64 #1, including the AAVS1 integration site, was confirmed via Sanger sequencing (Supplementary Table S2).

Then, we removed the lentiviral vector, containing Cas9v1-Myc, from cells expressing the full-length dCas9v2-VP64 #1, using the same AAV vector #3 as in Fig. 6. Lentivirus containing Cas9v1-Myc was removed from the genome in four out of 10 clones (40%), and immunoblot analyses of clones #1 and #2 (dCas9v2 (full)-VP64#1 + sgLenti #1 and #2) are shown in Supplementary Fig S6. Considering that reconstituted full-length dCas9v2-VP64 has no nuclease activity, we assume that the remaining Cas9v1-Myc protein, transcribed and translated from the Cas9v1-Myc gene before gene removal, worked to remove the lentivirus containing the Cas9v1-Myc gene itself. To confirm the ability of dCas9v2-VP64 to activate a specific promoter, we examined whether the modified sgRNA with bacteriophage coat protein MS2-binding RNA aptamers, targeting the IFNβ gene promoter site and MS2–P65 (NF-κB trans-activating subunit)-HSF1 (human heat shock factor 1) can activate IFNβ transcription (Supplementary Fig S9). The expression level of IFNβ transcription was monitored using ISRE-Luciferase activity via IFNAR, after transducing the indicated plasmids to express MS2-sgRNA and MS2–P65-HSF1, with an ISRE-Luc reporter plasmid. ISRE-Luciferase activity was significantly activated by IFNβ targeting (12.8- and 19.0-fold, respectively, compared to the control in clones #1 and #2, respectively); therefore, dCas9v2-VP64 effectively activates other genes.

### Reducing off-target effects using lentiviral Cas9 nickase

**Figure 7:**
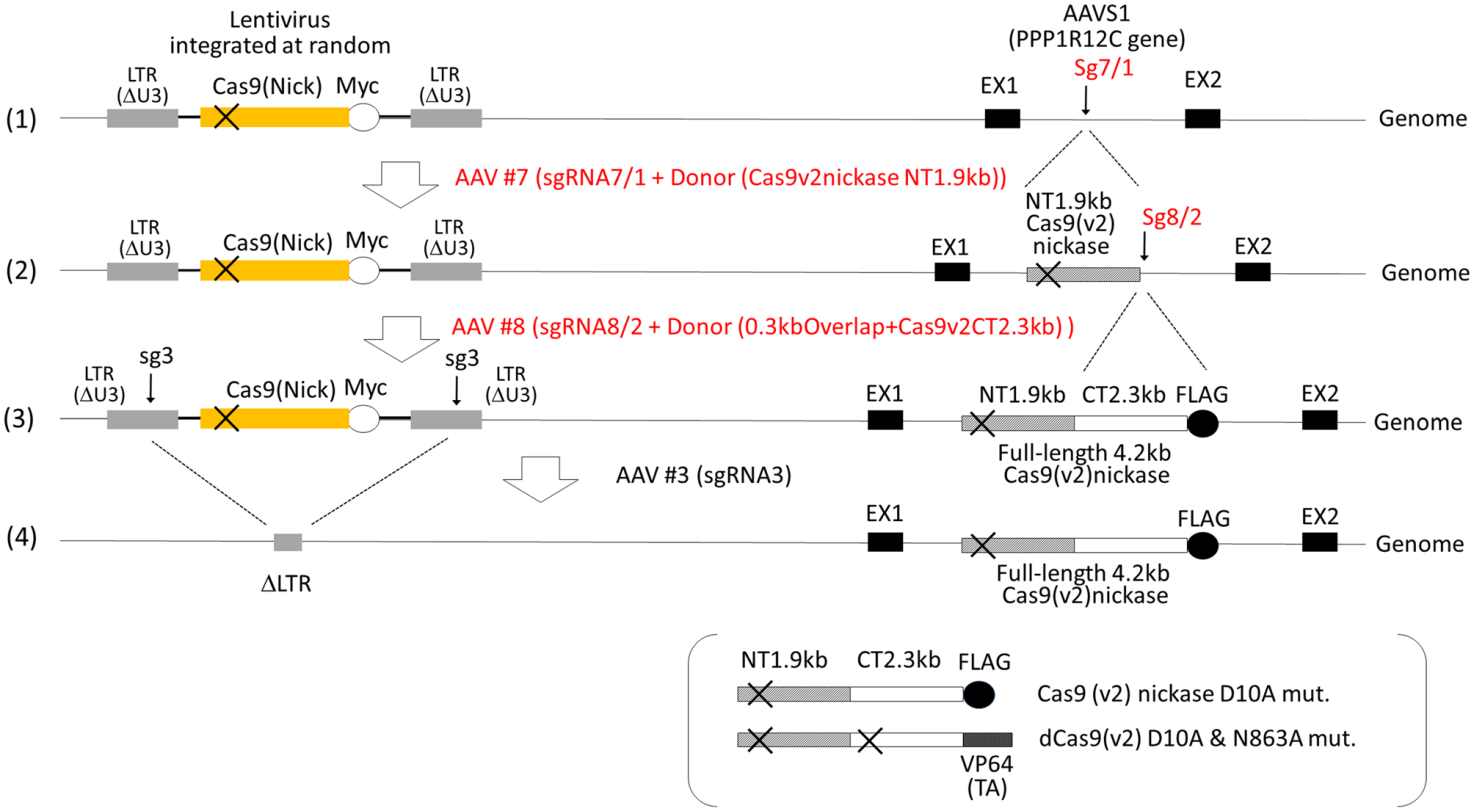
Schematic diagram of the four-step strategy using Lentiviral Cas9 (Nick)-Myc. (1) To reduce possible off-target effects, lentiviral Myc-tagged Cas9 nickase, termed Cas9 (Nick)-Myc (Supplementary Fig S10), was used in the first step. (2) AAV#7 (Supplementary Fig S11) was used to introduce DSB at the AAVS1 site via Cas9 (Nick)-Myc nickase. (3) AAV#8 (Supplementary Fig S12) was used to reconstitute the full-length 4.2 kb Cas9v2 nickase-FLAG. If AAV#9 (Supplementary Fig S13) was used instead of AAV#8, dead Cas9 (v2)-VP64 was produced, as shown in brackets. (4) Randomly integrated lentivirus, containing Cas9 (Nick)-Myc, was removed by AAV vector #3, with sgRNA3 targeting the long terminal repeat (LTR) of the lentivirus vector. sgRNAs targeting AAVS1, the 3′-end of the 1.9 kb Cas9v1 N-terminal fragment, and the LTR of the lentivirus are shown as sg7/1, sg8/2, and sg3, respectively.

The use of lentiviral Cas9 at the first step of the basic procedure stated above has potential off-target effects as well as a genetic risk due to its genomic integration. To reduce possible off-target effects caused by the lentiviral Cas9v1, during reconstitution of Cas9v2 nickase or dCas9v2-VP64 in the AAVS1 locus, we used lentiviral Cas9 nickase instead of wild-type Cas9v1 at the first step of the basic procedure (Fig. 7). As shown in Supplementary Fig S10A,B, Cas9 (Nick)-Myc was constructed by replacing the N-terminal of Cas9v1 with Cas9v2 nickase. This was performed at the unique BspHI restriction enzyme site, in order to differentiate the Cas9v2-FLAG and dCas9v2-VP64 integrated at the AAVS1 locus, from randomly integrated lentiviral Cas9(Myc)-Myc in the host genome. Cas9 (Nick)-Myc expression was confirmed by immunoblotting after hygromycin selection (Supplementary Fig S10C). Fortunately, there were possible Cas9 nickase cleavage sites at both the AAVS1 integration site and at the junction between the N-terminal of Cas9v2 and P2A (Supplementary Figs S11 and S12). To introduce the N-terminal Cas9v2 nickase with a D10A mutation into the AAVS1 locus, we constructed the AAV vector #7 by adding another sgRNA (sg7) to the AAV vector #5, for a DNA DSB via Cas9(Nick) (Supplementary Fig S11). To provide a double-cut donor, target sequences (T1) were also replaced with T7/1. The exact sequence of the AAV vector #7 is shown in Supplementary Table S1. The AAV vectors # 8 (Supplementary Fig S12) and #9 (Supplementary Fig S13) were constructed and used to reconstitute full-length Cas9v2 nickase-FLAG and dCas9v2-VP64, respectively. These sequences are listed in Supplementary Table S1.

### Sequential infection with AAV vectors without drug selection

**Figure 8:**
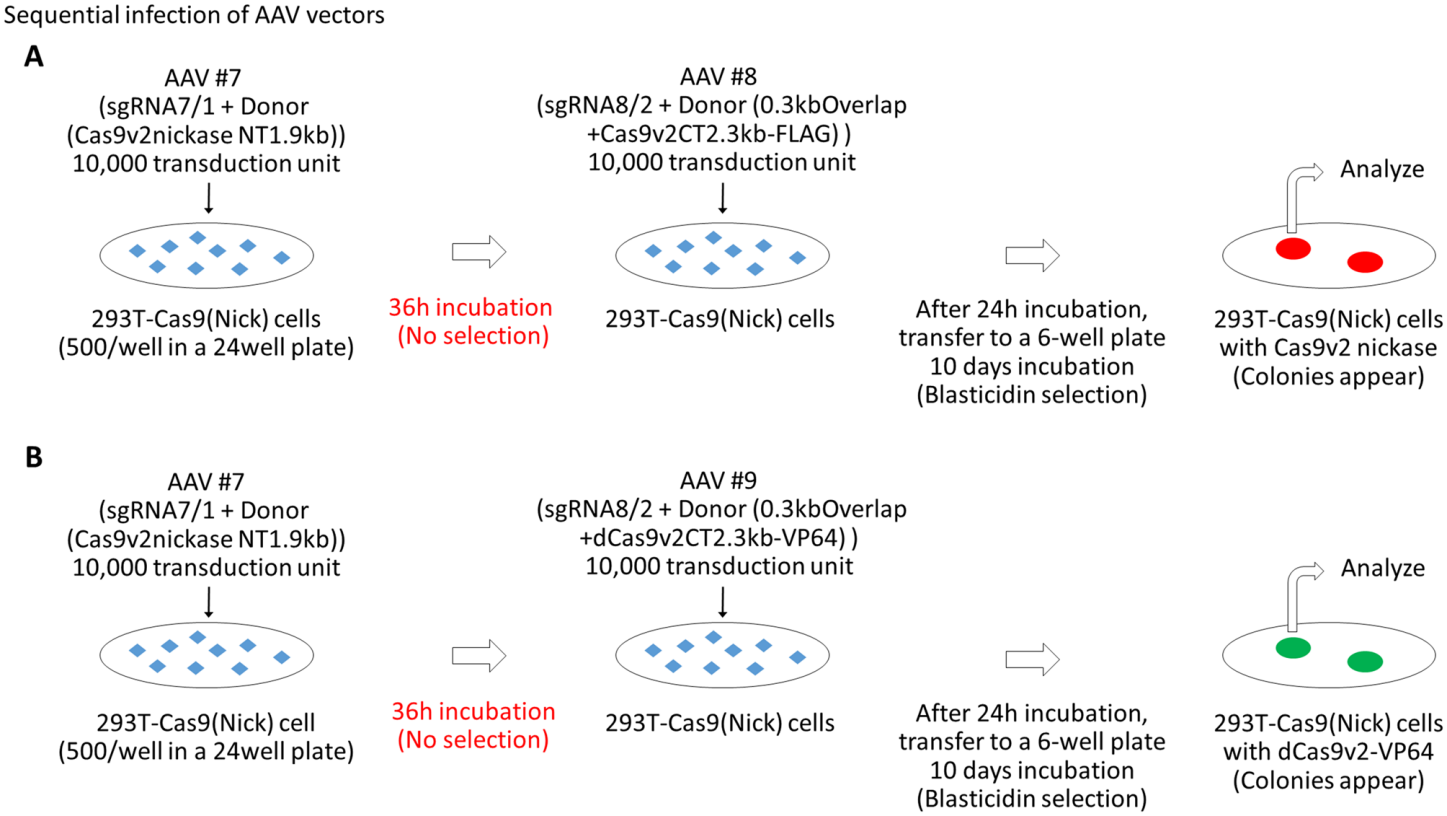
Sequential transduction procedure using AAV vectors. (**A**) According to the scheme shown in figure, 293 T-Cas9(Nick)-Myc cells (500/well) plated in a 24-well plate were incubated with 10,000 transduction units of AAV#7, which provided sgRNA targeting the AAVS1 site, and 1.9 kb Cas9v2 nickase N-terminal fragments (Supplementary Fig S11), for 36 h. Following medium aspiration, cells were sequentially incubated with 10,000 transduction units of AAV#8 (Supplementary Fig S12), for 24 h, to reconstitute full-length Cas9 (v2) nickase-FLAG. Cells were transferred to a 6-well plate, with blasticidin added two days after transfer. Colonies were collected after 10 days in the presence of blasticidin and analyzed using PCR and immunoblot. (**B**) 293 T-Cas9 (Nick)-Myc cells (500/well) were sequentially incubated with AAV#7 and AAV#9 (Supplementary Fig S13) to reconstitute full-length dCas9 (v2)-VP64. Blasticidin-resistant cells were analyzed using PCR and immunoblot.

**Figure 9:**
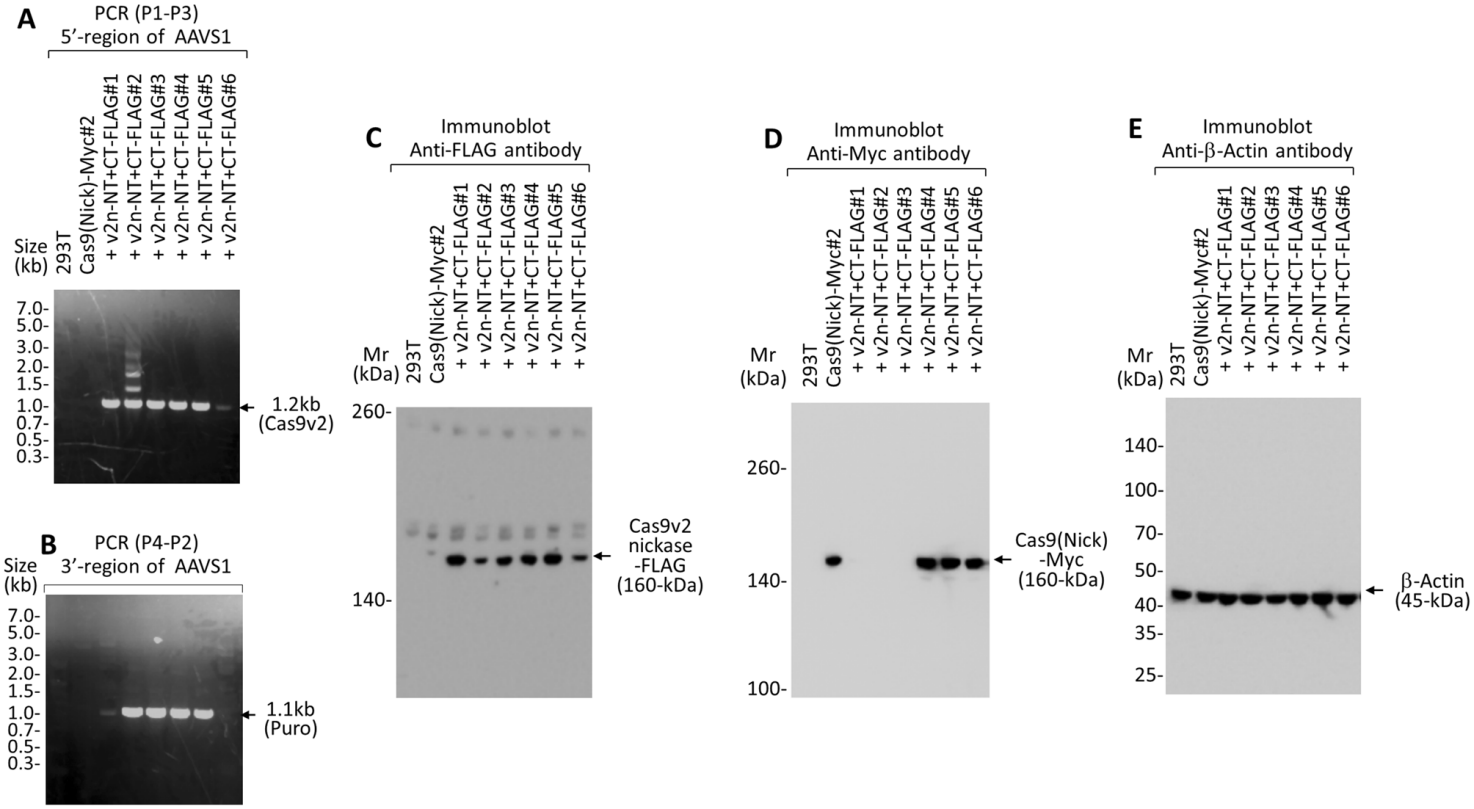
PCR and immunoblot analysis to confirm the reconstitution of full-length Cas9v2nickase-FLAG in the AAVS1 locus. (**A**,**B**) PCR analysis of genomic DNA prepared from HEK293T, HEK293T-Cas9(Nick)-Myc, and HEK293T-Cas9(Nick)-Myc + v2n-NT + CT-FLAG #1–#6 cells, after sequential infections of AAV vectors #7 and #8, as in Fig. 8A. v2n-NT, 1.9 kb Cas9v2 nickase-NT; CT-FLAG, 2.3 kb Cas9v2-CT-FLAG. Primers P1–P3 (Cas9v2), and P4–P2 (Puro), were used to examine the integration efficiency of the reconstituted full-length Cas9v2nickase-FLAG into the AAVS1 locus at the 5′- and 3′-regions. (**C**–**E**) Lysates obtained from the same cells used for the PCR analysis were analyzed using immunoblot, with the antibodies indicated. Arrows indicate reconstituted full-length Cas9v2nickase-FLAG (160 kDa), lentiviral Cas9 (Nick)-Myc (160 kDa), and β-actin (45 kDa).

**Figure 10:**
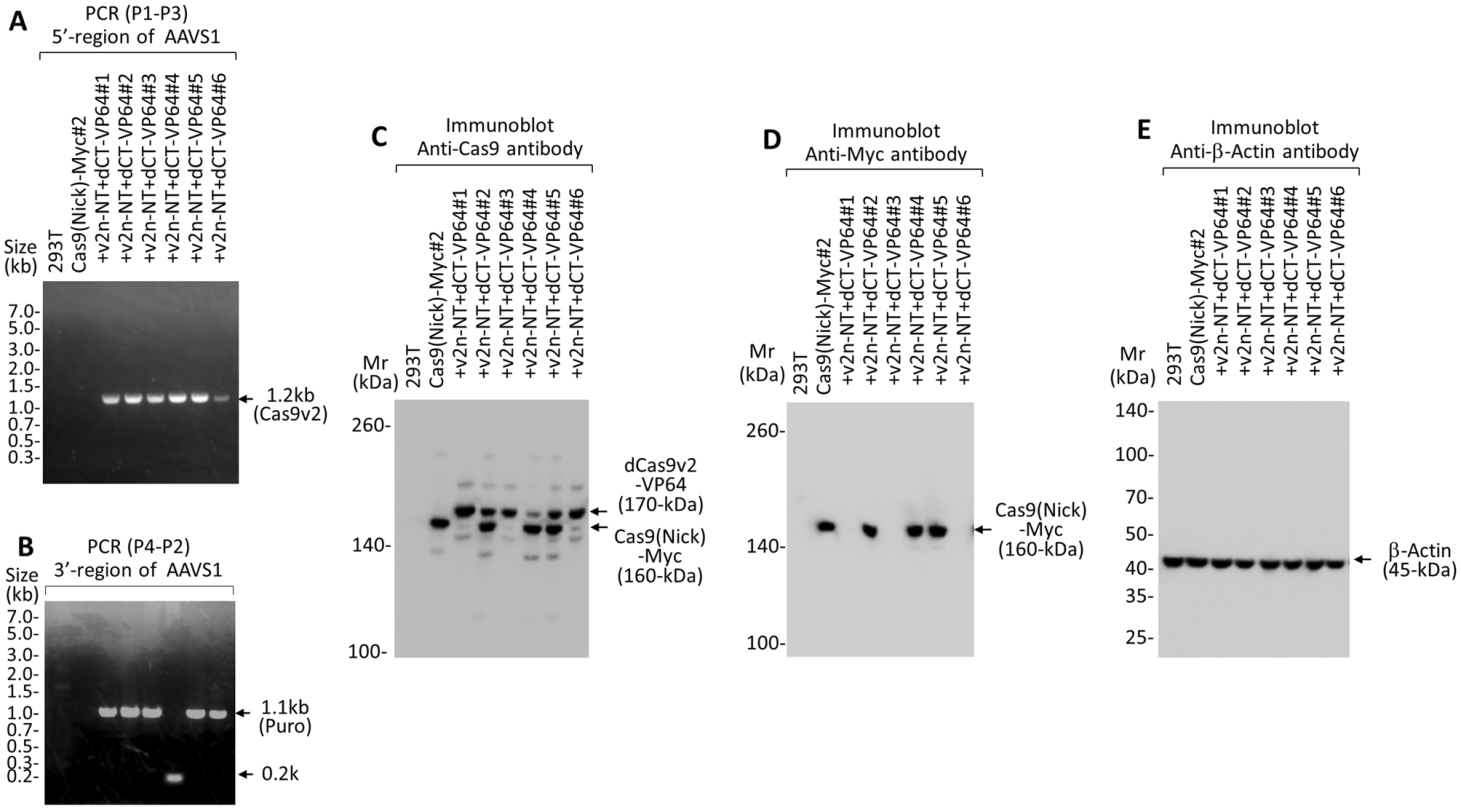
PCR and immunoblot analysis to confirm the reconstitution of full-length dCas9v2-VP64 in the AAVS1 locus. (**A**,**B**) PCR analysis of genomic DNA prepared from HEK293T, HEK293T-Cas9(Nick)-Myc, and HEK293T-Cas9(Nick)-Myc + v2n-NT + dCT-VP64 #1–#6 cells, after sequential infections of AAV vectors #7 and #9, as in Fig. 8B. v2n-NT, 1.9 kb Cas9v2 nickase-NT; dCT-VP64, 2.3 kb dCas9v2-CT-VP64. Primers P1–P3 (Cas9v2), and P4–P2 (Puro), were used to examine the integration efficiency of the reconstituted full-length dCas9v2-VP64 into the AAVS1 locus at the 5′- and 3′-regions. (**C**–**E**) Lysates obtained from the same cells used for the PCR analysis were analyzed using immunoblot, with the antibodies indicated. Arrows indicate reconstituted full-length dCas9v2-VP64 (170 kDa), lentiviral Cas9 (Nick)-Myc (160 kDa), and β-actin (45 kDa).

Genome restructuring commences soon after the introduction of sgRNA and donor dsDNA into 293 T-Cas9 (Nick)-Myc#2 cells. It suggested that Cas9v2 reconstitution from two fragments in the AAVS1 locus could be accomplished through sequential transductions with two AAV vectors within a short interval (24–36 h) and without drug selection between transductions. Following the method outlined in Fig. 8A, the full-length Cas9v2 nickase-FLAG was successfully reconstituted through sequential infections of the AAV vectors #7 and #8, within a short 36 h interval between transductions, and subsequent blasticidin selection (Fig. 9). Immunological and PCR analyses showed that full-length Cas9v2 nickase-FLAG was present at the AAVS1 locus in 83% (5/6) of blasticidin-resistant cells. Although all clones expressed the full-length Cas9v2 nickase-FLAG protein (Fig. 9C), clone #6 may carry a deletion in the 3′ region of the AAVS1 integration site including the puromycin gene (Fig. 9B). We also analyzed lentiviral Cas9 (Nick)-Myc. Unexpectedly, lentiviral Cas9 (Nick)-Myc proteins were not detected in clones #1, #2, and #3 (Fig. 9D). Considering that the Cas9 (Nick)-Myc genes were present in the genome of all the clones (Supplementary Fig S14), the sequential transductions to reconstitute Cas9v2 nickase-FLAG did not affect the lentiviral Cas9 (Nick)-Myc genome structure. Following the same procedure, dCas9v2-VP64 was also reconstituted through sequential transductions of the AAV vectors #7 and #9, within a short 36 h interval between transductions, and subsequent blasticidin selection (Fig. 8B). As shown in Fig. 10, full-length dCas9v2-VP64 was reconstituted successfully at the AAVS1 locus in all six clones (100%), although clone #4 was found to have a deletion around the right homology arm (Supplementary Fig S15B). Likewise, the lentiviral Cas9 (Nick)-Myc proteins were not detected in clones #1, #3, and #6 (Fig. 10D). All the clones had Cas9 (Nick)-Myc genes in their genome (Supplementary Fig S14). The overall transduction efficiency of these sequential transductions was approximately 3%, as an average of 15 colonies appeared from the initial 500 HEK293T-Cas9 (Nick)-Myc cells in both experiments.

It is difficult to use drug selection markers in vivo. To consider the possibility of in vivo use of this method, we measured the transduction efficiency at each step. For the first step of lentiviral introduction of Cas9 in the genome, we obtained Cas9 (Nick)-Myc-expressing colonies (1/10 and 1/10) in two independent experiments (10%) using the lentivirus containing Cas9 (Nick)-Myc (MOI = 2) without drug selection, as shown in Supplementary Fig S16. In the second step, however, we could not obtain any colonies expressing NT1.7k-Cas9v2 out of 30 colonies using a high concentration of AAV vector #1 (MOI = 20) without drug selection. In the third step, the cells containing the 1.9 kb N-terminal nickase fragment at the AAVS1 locus (Cas9v1-Myc + Cas9v2nickase-NT#9) were infected with high concentrations of the AAV vectors #2 or #6 (MOI = 20, each) without drug selection. The reconstitution rates of full-length Cas9v2nickase-FLAG and full-length dCas9v2-VP64 were 3/6 (50%) and 3/6 (50%), respectively. Supplementary Fig S17 shows the reconstituted full-length Cas9v2 nickase-FLAG with AAV #2 vector. In the fourth step, the removal rate of lentiviral Cas9v1 from the Cas9v1-Myc + Cas9v2(full)-FLAG#7 cells, without drug selection, was approximately 50% (#1: 3/6, #2: 5/9, and #3: 4/10 in the three independent experiments) using a high concentration of the AAV vector #3 (MOI = 20). Supplementary Fig S18 shows the data of #3 experiment without drug selection. We could not determine the transduction efficiency of the N-terminal Cas9v2 nickase fragment in the second step, as stated above. Considering that the induction efficiency of the sequential second and third infections was 3% and that the induction rate of the third step was 50%, the second step induction rate was simply expected to be 6%. If the third step induction rate is 100%, the second step induction rate is calculated to be 3%. However, sequential transductions might have a favorable effect on the second and third inductions. Therefore, we estimate the second step induction will be 3–6% without drug selection.

## Discussion

We proposed an effective method to introduce Cas9 into the AAVS1 locus using viral vectors. Stably expressed Cas9, especially Cas9 nickase, in the safe harbor can be used for precise gene editing. Considering that NHEJ, followed by DSB, is error-prone, precise genome editing using HDR is required, especially during in vivo use^5–7,20,21^. Although the first lentiviral induction of Cas9 (Nick)-Myc, using this method, was approximately 10% (MOI = 2), without drug selection (Supplementary Fig S16), the lentiviral induction rate could be increased using higher titers of virus. While the second induction efficiency of Cas9v2 N-terminal fragments was poor (speculated to be 3–6%), the induction rate of Cas9v2 C-terminal parts (the third step), was satisfactory without drug selection (about 50%) (Supplementary Fig S17). Sequential transfection data showed a Cas9 reconstitution rate of 3% from Cas9 (Nick)-Myc-expressing cells without drug selection (Figs. 8, 9, 10). Further, the overall Cas9 reconstitution rate was 0.3% of 293 T cells, given a lentiviral Cas9 (Nick)-Myc induction rate of 10%. The Cas9 (Nick)-Myc induction rate may be increased through higher concentrations of lentivirus (more than MOI = 2). Therefore, it is important to improve the efficacy of limiting N-terminal Cas9v2 induction in the second step. We speculate that the insertion of N-terminal Cas9v2 into one allele induces a structural change that may allow easier access to the target site, for the donor C-terminal Cas9v2, resulting in the high transduction efficiency of the third step. Some reagents and molecules have been reported to enhance HDR^7,27–29^. For example, CtIP-fused Cas9 was used to bring essential components to the Cas9-mediated DSB site, while monoavidin-fused Cas9 recruits biotinylated donor DNA to Cas9, thus enabling HDR^28–30^. Combining some of these methods with our system may further improve HDR rates.

The fourth step of our procedure focused on removing lentivirus containing Cas9v1-Myc; the removal rate was approximately 50%, without drug selection (Supplementary Fig S18). In addition, stable expression of Cas9v2-FLAG at the AAVS1 locus enables multiple attempts to remove lentivirus using the same or different sgRNA targeting LTR; thus, it was possible to repeat until all lentiviruses were removed^16,17^. As for the genetic risk, as each recombinant lentivirus is usually integrated into only one allele^31,32^, the destruction of a single tumor suppressor gene is not likely to be pathogenic. However, accidental activation of oncogenes, genes, or miRNAs critical for cell cycle or proliferation, via lentivirus insertion cannot be ruled out.

Alternative approaches to reduce the genetic risk associated with lentiviral integration into the genome may include transient expression of Cas9v1 using non-integrating lentiviral vector^13,14^. AAV vectors that express SaCas9 (small enough for AAV packaging)^10^, or split-Cas9 (N- and C-terminal parts are combined after translation)^11,12^ may be sufficient for Cas9v2 reconstitution at the AAVS1 locus. Figures 8, 9 and 10 show that integration of combined Cas9v2 nickase-FLAG or dCas9v2-VP64, at the AAVS1 locus, was completed within a couple of days via sequential transductions of the two AAV vectors (containing NT- and CT-Cas9v2 parts). Transient expression of Cas9(Nick)-Myc via non-integrating lentiviral vector, or SaCas9 via the AAV vector, appeared sufficient for the reaction as shown in Supplementary Fig S19. Consequently, the fourth step to remove the integrated lentivirus containing Cas9v1 could be omitted.

As for targeting cells for the integration of lentivirus carrying Cas9v1, used in the first step, many human cell types are susceptible to infection by the vesicular stomatitis virus G protein (VSVG)-pseudotyped recombinant lentiviruses^33^. However, the broad VSVG tropism can be controlled through the insertion of a 49-amino-acid peptide (echistatin), or a single-chain variable fragment (scFv) at the N-terminal, or some specific VSVG sites, thus directing it to cells expressing integrin αVβ3 or the molecule against scFv^34,35^. If a lentivirus carrying Cas9v1 is pseudotyped with the modified VSVG, specific cells could be targeted to express Cas9v1. That enables the subsequent Cas9v2 integration at the AAVS1 locus only in the targeted cells.

Thus, the merits of our method over existing single-vector technologies for gene therapy are as follows: (1) stable and robust Cas9v2 expression in the AAVS1 locus facilitates precise gene editing when using HDR. (2) The availability of various types of modified Cas9 that exceed the AAV vector packaging capacity, including Cas9 nickase, to minimize off-target effects (Supplementary Fig S19). (3) Using non-integrating lentiviral vector to express Cas9 nickase transiently, or AAV-SaCas9 for the first step, could minimize genetic risk and save the fourth step (Supplementary Fig S19). (4) Targeting cells for integration could be controlled by pseudotyped with modified VSVG. (5) The high lentivirus removal rate (Supplementary Fig S18); sgRNA targeting LTR could be used to remove the lentivirus if illegitimate integration caused abnormal cell growth. This not only provides safety when using lentivirus for gene therapy but may also be used for the removal of human immunodeficiency virus (HIV), human T-cell leukemia virus type 1 (HTLV1), integrated in acquired immune deficiency syndrome (AIDS) or adult T-cell leukemia/lymphoma.

In order to apply our method in vivo, we have to overcome the overall low reconstitution rate of Cas9 in the AAVS1 locus without drug selections as stated above. In addition, the presence of neutralizing antibodies against wild-type AAVs in the host body deters the therapeutic use of AAV vectors^8^. There are concerns that the host immune reaction to the capsid of the AAV #1 may prevent the subsequent transduction of the AAV #2 and #3, and that the stable and robust expression of foreign protein Cas9 (not only full-length Cas9 but also N-terminal Cas9 that was not successfully reconstituted) may induce unwanted immune reactions^36^. Targeting specific genes such as the STING-STAT6 axis, however, might prevent these unwanted immune responses^37–39^. Sequential transductions of AAVs within short intervals, as shown in Figs. 8, 9 and 10, might conclude the gene therapy process before the induction of neutral antibodies against previous AAVs. However, we must take into account the fatal risk of using high concentrations of AAV vectors in vivo^40^.

## Methods

### Cell lines and cell culture

Human embryonic kidney HEK293T and LENTI-X 293 T cells (Takara Bio Inc. #632180) were cultured in Dulbecco’s modified Eagle’s medium supplemented with 10% fetal bovine serum. To generate stable cell lines, we used 0.3 mg/mL hygromycin B (Wako #084-07681), 2.5 μg/mL puromycin (Nacalai #14861-71), or 32 μg/mL blasticidin S (Wako #026-18711) for at least two weeks unless otherwise described.

### Virus constructs

The SIN-type lentiviral vector pLVSIN-EF1a Hyg (#6185) and AAV2 serotype vector pAAV-CMV (#6230) were purchased from Takara Bio Inc. pCas-Guide (#GE10002) was purchased from OriGene Tech. Inc. lentiCRISPR v2 was a gift from Feng Zhang (Addgene plasmid #52961; http://n2t.net/addgene:52961)^19^. lentiSAMv2 was also a gift from Feng Zhang (Addgene plasmid #75112; http://n2t.net/addgene:75112)^26^. The lentivirus pLVSIN was modified to express Cas9v1 (SpCas9 from pCas-Guide)-Myc and hygromycin resistance gene (Hygro) connected to the self-cleaving peptide T2A under a CMV promoter. Cas9(Nick)-Myc was constructed by replacing N-terminal Cas9v1 with Cas9v2 nickase at the unique BspHI restriction enzyme site, as shown in Supplementary Fig S10. After infecting 293 T cells with a series of viral solution dilutions, the lentiviral titer (transduction unit/ml) was determined from the number of colonies that survived for ten days in the presence of 0.3 mg/mL hygromycin B.

An AAV vector #1 targeting the AAVS1 locus (T1) was constructed by placing the AAVS1-specific sgRNA1 (sg1) and several elements by the AAVS1-driven transcript flanking the homology arms between the ITRs. These elements are SA-T2A (a self-cleaving peptide), 1.9 kb N-terminal Cas9v2 fragment T2A (a self-cleaving peptide)-puromycin resistance gene, and a polyadenylation (pA) sequence. The targeting sequences (T1) were added adjacent to the homology arms to enable HDR by creating a double-cut donor. The sequences are shown in Supplementary Table S1.

The AAV vector #2 sequence is also listed in Supplementary Table S1. sgRNA2 (sg2) targets the junction (T2) between the 3′-end of 1.9 kb N-terminal Cas9v2 and the 5′-end of P2A (a self-cleaving peptide). To reconstitute the full-length of Cas9v2, we overlapped the 0.3 kb region between the 1.9 kb N-terminal and 2.3 kb C-terminal region of Cas9v2. The 0.3 kb overlap works as the left arm in HDR. As for the right arm, the P2A and N-terminal 0.3 kb fragment of puromycin flank the T2 targeting sequence and the ITR to provide a double-cut donor.

The AAV vector #3 targets the LTR of the lentiviral vector by a pair of sgRNAs (sg3-1 and sg3-2) with a 16 bp offset for DSB by nickase Cas9 and wild-type Cas9, as shown in Supplementary Table S1. The AAV vector #4 targeting exon1 of IFNAR1 (T4) was constructed by placing a pair of sgRNAs (sg4-1 and sg4-2) with a 7 bp offset for DSB by nickase Cas9 and wild-type Cas9, and placing the hygromycin-resistance gene between the ITRs. The AAV #5 was the same as AAV #1 except for the D10A mutation (GAC → GCC) in the 1.7 kb N-terminal Cas9v2, and AAV #6 was the same as AAV #2 except for the N863A mutation (AAC → GCC) in the 2.3 kb C-terminal Cas9v2 with VP64 transcriptional activator. AAV vector #7 was constructed by adding another sgRNA (sg7) to the AAV vector #5 and replacing the T1 target sequence with T7/1, as shown in Supplementary Fig S11. The AAV vectors #8 (Supplementary Fig S12) and #9 (Supplementary Fig S13) were also constructed by adding another sgRNA (sg8) to AAV vectors #2 and #6, respectively, and replacing the T2 target sequence with T8/2. The exact sequences of AAV vectors #7, #8, and #9 are listed in Supplementary Table S1.

After infecting 293 T cells with a series of diluted viral solutions, the AAV viral titer (transduction unit/ml) of vectors #3 and #4 was directly determined via the number of colonies that survived for ten days in the presence of 2.5 μg/mL puromycin or 0.3 mg/mL hygromycin B. The bioassay is not suitable for the AAV vectors #1, #2, #5, #6, #7, #8, or #9, because the drug resistance markers are activated only when they are integrated into the designated sites. So, we used real-time PCR, with specific primers (P11-P12 in Supplementary Table S3) to first measure the physical amount of puromycin gene in each virus solution, after residual DNA fragments were removed from the virus solution by incubation with an anion-exchange resin in 0.3 M NaCl, and 20 mM Tris, pH7.0 (Q-FAST FLOW, Pharmacia). Next, the physical amount of puromycin gene was used to estimate the biological viral titer of each virus solution (transduction units/mL), referring to the relationship between the physical amount of puromycin gene of the AAV vector #3 virus solution determined by real-time PCR and the biological viral titer of AAV #3 virus solution.

### Cell transduction

The SIN-type lentivirus vector was co-transfected with ViraPower lentiviral packaging mix (Invitrogen #K497500) into Lenti-X 293 T cells (Takara #632180) using Lipofectamine 2000 transfection reagent (Thermo Fisher Scientific #11668027). After 3 days, the supernatant containing recombinant virus particles was concentrated using Lenti-X concentrator (Takara Bio #631231) and used for cell infection (MOI = 0.1–2) in the presence of 8 μg/mL Polybrene (Nakalai #12996-81). Each AAV vector was co-transfected with pHelper and pRC-mi342 vectors (Takara #6234 for AAV2) into HEK293T cells using the Lipofectamine 2000 transfection reagent. After three days, the recombinant AAV particles were extracted with AAVpro Extraction Solution (Takara #6235) according to the manufacturer’s instructions and used for cell transduction.

For sequential transduction, 293 T-Cas9 (Nick)-Myc cells (500/well) plated in a 24-well plate were incubated with 10,000 transduction units of AAV #7, which provided sgRNA targeting the AAVS1 site, along with 1.9 kb Cas9v2 nickase N-terminal fragments, for 36 h. Following medium aspiration, cells were sequentially incubated with 10,000 transduction units of AAV #8 or AAV #9 for 24 h, to reconstitute the full-length Cas9(v2) nickase-FLAG or dCas9(v2)-VP64, respectively. Cells were transferred to a 6-well plate, and blasticidin was added two days after transfer. Colonies were collected after 10 days in the presence of 32 μg/mL blasticidin S and analyzed using PCR and immunoblot.

### Genomic DNA isolation and PCR

Genomic DNA was extracted from semi-confluent cells plated in 24-well plates. After 1% Triton X-100-mediated extraction of cytosolic content, samples were treated with 180 μg/mL proteinase K (Nacalai #15679-06) for 24 h in a buffer containing 0.5% SDS. Samples were treated with phenol/chloroform/isoamyl alcohol (25:24:1), precipitated with ethanol, and dissolved in TE after 70% ethanol wash. Genomic DNA (100 ng each) was subjected to PCR using the specific primers listed in Supplementary Table S3, and KOD-FX-NEO DNA Polymerase (TOYOBO #KFX-201) on a LightCycler 1.5 (ABI). The general reaction conditions were 94 °C for 2 min followed by 35 cycles of 10 s of denaturation at 98 °C, 10 s of annealing at 62 °C, and 1 min of extension at 68 °C unless otherwise described^41^. The PCR products were separated on a 1% agarose gel. DNA fragments were purified, and the sequences were directly determined by Sanger sequencing.

### Immunoblot analysis

Cell lysates were extracted from semi-confluent cells plated in 24-well plates using GLO lysis buffer (Promega #E2661). The protein concentration of each cell lysate was measured with BCA (bicinchoninic acid) protein assay, and the 10 μg aliquot was separated by 5–12.5% SDS-PAGE and transferred to poly vinylidene di-fluoride (PVDF) membranes. Specific proteins were detected using an anti-Cas9 polyclonal antibody (Takara Bio #632607), anti-Myc-tag (9B11) mouse monoclonal antibody (Cell Signaling Technology #2276S), anti-FLAG M2 monoclonal antibody (Sigma-Aldrich #F1804), or anti-β-actin mouse monoclonal antibody (Santa-Cruz #SC-47778) and were used at 1:500–3000 dilution. They were visualized with HRP-conjugated anti-rabbit or anti-mouse IgG antibody and ECL reagent^41^.

## Supplementary information

Supplementary Information.

Supplementary Tables.
