# Supplementary material for: Efficient viral delivery of Cas9 into human safe harbor
Source: Sci Rep. 2020 Dec 8;10:21474. doi: 10.1038/s41598-020-78450-8 (PMC7722726; doi:10.1038/s41598-020-78450-8)

[Supplementary Information]

Efficient viral delivery of Cas9 into human safe harbor

Hideki Hayashi[1]*, Yoshinao Kubo[2], Mai Izumida[2], and Toshifumi Matsuyama[3].

[1]Medical University Research Administrator, Nagasaki University School of Medicine, Nagasaki, Japan

[2]Department of Clinical Medicine, Institute of Tropical Medicine, Nagasaki University, Nagasaki, Japan

[3]Department of Cancer Stem Cell Biology, Graduate School of Biomedical Sciences, Nagasaki University, Nagasaki, Japan

*Corresponding author
E-mail: hhayashi@nagasaki-u.ac.jp

Methods

Cell lines and cell culture

Human embryonic kidney HEK293T and LENTI-X 293T cells (Takara Bio Inc. #632180) were cultured in Dulbecco's modified Eagle's medium supplemented with 10% fetal bovine serum. To generate stable cell lines, we used 0.3 mg/mL hygromycin B (Wako #084-07681), 2.5 μg/mL puromycin (Nacalai #14861-71), or 32 μg/mL blasticidin S (Wako #026-18711) for at least two weeks unless otherwise described.

Virus constructs

The SIN-type lentiviral vector pLVSIN-EF1a Hyg (#6185) and AAV2 serotype vector pAAV-CMV (#6230) were purchased from Takara Bio Inc. pCas-Guide (#GE10002) was purchased from OriGene Tech. Inc. lentiCRISPR v2 was a gift from Feng Zhang (Addgene plasmid #52961; http://n2t.net/addgene:52961). lentiSAMv2 was also a gift from Feng Zhang (Addgene plasmid #75112; http://n2t.net/addgene:75112). The lentivirus pLVSIN was modified to express Cas9v1 (SpCas9 from pCas-Guide)-Myc and hygromycin resistance gene (Hygro) connected to the self-cleaving peptide T2A under a CMV promoter. Cas9(Nick)-Myc was constructed by replacing N-terminal Cas9v1 with Cas9v2 nickase at the unique BspHI restriction enzyme site, as shown in Supplementary Fig S10. After infecting 293T cells with a series of viral solution dilutions, the lentiviral titer (transduction unit/ml) was determined from the number of colonies that survived for ten days in the presence of 0.3 mg/mL hygromycin B.

An AAV vector #1 targeting the AAVS1 locus (T1) was constructed by placing the AAVS1-specific sgRNA1 (sg1) and several elements by the AAVS1-driven transcript flanking the homology arms between the ITRs. These elements are SA-T2A (a self-cleaving peptide), 1.9 kb N-terminal Cas9v2 fragment T2A (a self-cleaving peptide)-puromycin resistance gene, and a polyadenylation (pA) sequence. The targeting sequences (T1) were added adjacent to the homology arms to enable HDR by creating a double-cut donor. The sequences are shown in Supplementary Table S1.

The AAV vector #2 sequence is also listed in Supplementary Table S1. sgRNA2 (sg2) targets the junction (T2) between the 3'-end of 1.9 kb N-terminal Cas9v2 and the 5'-end of P2A (a self-cleaving peptide). To reconstitute the full-length of Cas9v2, we overlapped the 0.3 kb region between the 1.9 kb N-terminal and 2.3 kb C-terminal region of Cas9v2. The 0.3 kb overlap works as the left arm in HDR. As for the right arm, the P2A and N-terminal 0.3 kb fragment of puromycin flank the T2 targeting sequence and the ITR to provide a double-cut donor.

The AAV vector #3 targets the LTR of the lentiviral vector by a pair of sgRNAs (sg3-1 and sg3-2) with a 16bp offset for DSB by nickase Cas9 and wild-type Cas9, as shown in Supplementary Table S1. The AAV vector #4 targeting exon1 of IFNAR1 (T4) was constructed by placing a pair of sgRNAs (sg4-1 and sg4-2) with a 7bp offset for DSB by nickase Cas9 and wild-type Cas9, and placing the hygromycin-resistance gene between the ITRs. The AAV #5 was the same as AAV #1 except for the D10A mutation (GAC→GCC) in the 1.7 kb N-terminal Cas9v2, and AAV #6 was the same as AAV #2 except for the N863A mutation (AAC→GCC) in the 2.3 kb C-terminal Cas9v2. AAV vector #7 was constructed by adding another sgRNA (sg7) to the AAV vector #5 and replacing the T1 target sequence with T7/1, as shown in Supplementary Fig S11. The AAV vectors #8 (Supplementary Fig S12) and #9 (Supplementary Fig S13) were also constructed by adding another sgRNA (sg8) to AAV vectors #2 and #6, respectively, and replacing the T2

target sequence with T8/2. The exact sequences of AAV vectors #7, #8, and #9 are listed in Supplementary Table S1.

After infecting 293T cells with a series of diluted viral solutions, the AAV viral titer (transduction unit/ml) of vectors #3 and #4 was directly determined via the number of colonies that survived for ten days in the presence of 2.5 μg/mL puromycin or 0.3 mg/mL hygromycin B. The bioassay is not suitable for the AAV vectors #1, #2, #5, #6, #7, #8, or #9, because the drug resistance markers are activated only when they are integrated into the designated sites. So, we used real-time PCR, with specific primers (P11-P12 in Supplementary Table S3) to first measure the physical amount of puromycin gene in each virus solution, after residual DNA fragments were removed from the virus solution by incubation with an anion-exchange resin in 0.3M NaCl, and 20mM Tris, pH7.0 (Q-FAST FLOW, Pharmacia). Next, the physical amount of puromycin gene was used to estimate the biological viral titer of each virus solution (transduction units/mL), referring to the relationship between the physical amount of puromycin gene of the AAV vector #3 virus solution determined by real-time PCR and the biological viral titer of AAV #3 virus solution.

Cell transduction

The SIN-type lentivirus vector was co-transfected with ViraPower lentiviral packaging mix (Invitrogen #K497500) into Lenti-X 293T cells (Takara #632180) using Lipofectamine 2000 transfection reagent (Thermo Fisher Scientific #11668027). After 3 days, the supernatant containing recombinant virus particles was concentrated using Lenti-X concentrator (Takara Bio #631231) and used for cell infection (MOI=0.1-2) in the presence of 8 μg/mL Polybrene (Nakalai #12996-81). Each AAV vector was co-transfected with pHelper and pRC-mi342 vectors (Takara #6234 for AAV2) into HEK293T cells using the Lipofectamine 2000 transfection reagent. After three days, the recombinant AAV particles were extracted with AAVpro Extraction Solution (Takara #6235) according to the manufacturer's instructions and used for cell transduction.

For sequential transduction, 293T-Cas9 (Nick)-Myc cells (500/well) plated in a 24-well plate were incubated with 10,000 transduction units of AAV #7, which provided sgRNA targeting the AAVS1 site, along with 1.9 kb Cas9v2 nickase N-terminal fragments, for 36 h. Following medium aspiration, cells were sequentially incubated with 10,000 transduction units of AAV #8 or AAV #9 for 24 h, to reconstitute the full-length Cas9(v2) nickase-FLAG or dCas9(v2)-VP64, respectively. Cells were transferred to a 6-well plate, and blasticidin was added two days after transfer. Colonies were collected after 10 days in the presence of 32 μg/mL blasticidin S and analyzed using PCR and immunoblot.

Luciferase assay

To investigate the function of reconstituted dCas9v2-VP64 from sgLenti #1 and #2 cells (Supplementary Fig S9), we examined activation of endogenous interferon (IFN) β gene promoter in cells (1×10^4/well) on a 48-well plate. Cells were transfected with 10 ng of 4xISRE-luciferase plasmid DNA, 10 ng MS2-P65-HSF1 plasmid DNA, and 10 ng MS2-sgRNA DNA, targeting the IFNβ gene promoter site or control, with 0.2 μL of lipofectamine 2000 transfection reagent (Thermo Fisher Scientific #11668027). Luciferase activity was determined by the addition of Steady-Glo luciferase substrate 36 h post-transfection. The sequences of MS2-sgRNA DNA, targeting the IFNβ gene promoter site or control are listed in Supplementary Table S4.

Genomic DNA isolation and PCR

Genomic DNA was extracted from semi-confluent cells plated in 24-well plates. After 1% Triton X-100-mediated extraction of cytosolic content, samples were treated with 180 μg/mL proteinase K (Nacalai #15679-06) for 24 h in a buffer containing 0.5% SDS. Samples were treated with phenol/chloroform/isoamyl alcohol (25:24:1), precipitated with ethanol, and dissolved in TE after 70% ethanol wash. Genomic DNA (100 ng each) was subjected to PCR using the specific primers listed in Supplementary Table S3, and KOD-FX-NEO DNA Polymerase (TOYOBO #KFX-201) on a LightCycler 1.5 (ABI). The general reaction conditions were 94°C for 2 min followed by 35 cycles of 10 s of denaturation at 98°C, 10 s of annealing at 62°C, and 1 min of extension at 68°C unless otherwise described. The PCR products were separated on a 1% agarose gel. DNA fragments were purified, and the sequences were directly determined by Sanger sequencing.

DNA sequence analysis

The precise genomic structure of some established cells was analyzed via direct sequencing of amplified PCR fragments from purified genomic DNA. The results are summarized in Supplementary Table S2.

For the IFNAR1 gene, genomic DNA from each clone was used as a PCR template to amplify the region around exon 1, with P9–P10 primers (Supplementary Fig S4). Primer sequences are listed in Supplementary Table S3. Amplified DNA fragments (usually heterogeneous around 0.4 kb) were cut from an agarose gel, purified using a GENE CLEAN kit (Funakoshi #1102-200), and cloned into a pcDNA vector plasmid. Plasmid DNA sequences (more than four clones from one cell line) were determined for structure analysis.

Immunoblot analysis

Cell lysates were extracted from semi-confluent cells plated in 24-well plates using GLO lysis buffer (Promega #E2661). The protein concentration of each cell lysate was measured with BCA (bicinchoninic acid) protein assay, and the 10 μg aliquot was separated by 5–12.5% SDS-PAGE and transferred to PVDF (Poly Vinylidene Di-Fluoride) membranes. Specific proteins were detected using an anti-Cas9 polyclonal antibody (Takara Bio #632607), anti-Myc-tag (9B11) mouse monoclonal antibody (Cell Signaling Technology #2276S), anti-FLAG M2 monoclonal antibody (Sigma-Aldrich #F1804), or anti-β-actin mouse monoclonal antibody (Santa-Cruz #SC-47778) and were used at 1:500–3,000 dilution. They were visualized with HRP-conjugated anti-rabbit or anti-mouse IgG antibody and ECL reagent.

Statistical analysis

Quantitative data were analyzed using a 2-tailed Student's *t*-test. The n values of each analysis are presented in the Figure legends. A value of $p<0.01$ was considered statistically significant.

Legends for Supplementary Figures

Supplementary Fig S1

Expression of Cas9v1-Myc in HEK293T cells. Human embryonic kidney (HEK) 293T cells were infected with a lentivirus to stably express Cas9v1-Myc. After selection with hygromycin for two weeks, hygromycin-resistant colonies were collected, and cell lysates were analyzed using immunoblotting, with an antibody against Cas9 following 6% SDS-PAGE. The arrow indicates Cas9v1-Myc (160 kDa).

Supplementary Fig S2

Structures of the two alleles of N-terminal Cas9v2 clone #8 in Fig 3A. A. According to direct sequences of the 4.5 kb and 4.2 kb PCR products from the N-terminal Cas9v2 clone #8 (Fig 3A), the 4.5 kb producing allele was as designed, and was the same as clone #4. **B**. The 4.2 kb producing allele was found to have an insertion and a deletion around the right arm. EX, exon; LA & RA, left and right homology arms; SA, splicing acceptor; T2A & P2A: *Thosea asigna* virus and porcine teschovirus-1 2A self-cleaving peptides; Puro, puromycin resistance gene; pA, a polyadenylation signal sequence; U6, U6 RNA polymerase III promoter. Primers P1-P2 targeted outside the homology arms to detect the insertion in the AAVS1 locus. The PCR primer sequences are listed in Supplementary Table S3.

Supplementary Fig S3

AAV vector #4 construct to knockout IFNAR1. A. AAV vector #4 targeting exon1 of the interferon-α/β receptor (IFNAR1) (T4) was constructed to contain a pair of sgRNAs (sg4-1 and sg4-2) with 7bp offset under U6 promoters to knock out IFNAR1 by NHEJ, and a hygromycin resistance gene under a PGK promoter was added for selection. **B.** Genomic structure around the exon1 of IFNAR1 gene in HEK293T-Cas9v2-FLAG+sgLenti#10 cells (Fig 6). ITR, inverted terminal repeat; P_{PGK}, PGK phosphoglycerate kinase promoter; Hygro, hygromycin resistance gene. The sgRNA sequences are shown in Supplementary Table S1.

Supplementary Fig S4

DNA sequence analysis of the IFNAR1 gene after sgRNA treatment. HEK293T-Cas9v2-FLAG+sgLenti#10 cells were infected with AAV #4 vector , as in Supplementary Fig S3. Genomic DNA from the hygromycin-resistant cells expressing sgIFNAR1 (independent two clones #1 and #3), was amplified using PCR, with P9-P10 primers. The amplified fragments (approximately 0.4 kb) were cloned into a plasmid, and the DNA sequences were determined by Sanger sequencing. **A**. The DNA sequence around IFNAR1 exon 1 (the translational region in upper case, with the corresponding amino acid sequence below) is shown on the top panel, indicating a sgRNA pair (sg4-1 and sg4-2) targeting sites with a 7 bp offset. Clones #1 and #3 had heterogeneous alleles. **B**. One allele of clone #1, with an insertion (shown in *italics*), and deletion (shown with hyphens), resulted in a loss of the IFNAR1 translation start site. Another allele of clone #1, with a deletion (shown with hyphens), resulted in a loss of the IFNAR1 translation start site. **C**. One allele of clone #3, with an insertion (shown in *italics*), and deletion (shown with hyphens), resulted in a frameshift (shown in *italics*) of IFNAR1. Another allele of clone #3 had a loss of the IFNAR1 translation start site because of a deletion (shown with hyphens).

Supplementary Fig S5

AAV vector #5 construct to introduce N-terminal 1.9 kb Cas9v2 nickase into the AAVS1 locus. A. The 1.9 kb N-terminal Cas9v2 with D10A mutation indicated as X (AAV vector #5) was used instead of AAV vector #1 in Fig 2, to introduce the N-terminal Cas9v2 nickase into the AAVS1 site. T1, AAVS1 target site with PAM (protospacer adjacent motif); sg1, AAVS1-specific sgRNA1. The exact sequence is shown in Supplementary Table S1. **B** shows the genome structure after integration of the donor by HDR. Immunoblot analyses of the puromycin-resistant HEK-Cas9v1-Myc#2+Cas9v2nickase-NT#7 and #9 cells were shown in Supplementary Fig S6. Primers (P1-P2) for PCR targeted outside of the homology arms. Primers (P1-P3) and (P4-P2) were used to detect the N-terminal Cas9 nickase and the Puro genes, respectively. The primer sequences are listed in Supplementary Table S3.

Supplementary Fig S6

Immunoblot analysis of two independent clones at each step of all procedures. After 7% SDS-PAGE, cell lysates, prepared from the indicated cells, were analyzed by immunoblot, with an anti-Cas9 antibody (**A**), an anti-Myc antibody (**C**), and an anti-FLAG antibody (**D**). 12.5% SDS-PAGE was used was used for immunoblotting with an anti-ß-actin antibody, (**B**). 293T, Cas9v1-Myc#2, #3 (Supplementary Fig S1); Cas9v1-Myc#2+Cas9v2-NT#4, #5 (Fig 3); Cas9v1-Myc#2+Cas9v2(full)-FLAG#7, #8 (Fig 5); Cas9v2(full)-FLAG#7 + sgLenti#7, #10 (Fig 6); Cas9v1-Myc#2+Cas9v2nickase-NT#7, #9; Cas9v1-Myc#2+Cas9v2nickase(full)-FLAG#3, #4; Cas9v2nickase(full)-FLAG#3+sgLenti#4, #5; Cas9v1-Myc#2+dCas9v2(full)-VP64#1, #2; dCas9v2(full)-VP64#1+ sgLenti#1, #2. Arrows indicate the reconstituted full-length dCas9v2-VP64 (170 kDa), full-length Cas9v2-FLAG, or Cas9v1-Myc (160 kDa), the 1.9 kb N-terminal Cas9v2 (70 kDa), and β-actin (45 kDa). β-actin serves as the loading control.

Supplementary Fig S7

Reconstitution of full-length Cas9v2-FLAG nickase in the AAVS1 locus. A. AAV vector#2 was used to reconstitute full-length Cas9v2-FLAG nickase in the AAVS1 locus. The 2.6 kb C-terminal fragment has the overlapping 0.3 kb region with the 1.9 kb N-terminal Cas9v2 nickase fragment to reconstitute full-length Cas9v2-FLAG nickase by HDR. T2, the target site of the junction between the 3'-end of 1.9 kb N-terminal Cas9v2 nickase and the 5'-end of P2A (a self-cleaving peptide) with PAM; sg2, the target site T2-specific sgRNA2; Bsd, blasticidin resistance gene. **B** shows the genome structure after integration of the donor by HDR. Primers (P5-P6) were set to detect the Bsd genes in the AAVS1 locus after HDR. Immunoblot analyses of the blasticidin-resistant HEK-Cas9v1-Myc#2+Cas9v2nickase(full)-FLAG#3 and #4 cells were shown in Supplementary Fig S6. *The puromycin resistant gene is not translated to the protein because of the stop codon following the blasticidin resistance gene.

Supplementary Fig S8

AAV vector #6 construct to reconstitute full-length dCas9-VP64 in the AAVS1 locus. A. AAV vector #6 is designed to reconstitute full-length Cas9v2 as a double cut donor. T2 is the target site of the junction between the 3'-end of 1.9 kb N-terminal Cas9v2nickase and the 5'-end of P2A (a self-cleaving peptide) with PAM. The 2.6 kb C-terminal fragment of dCas9v2 has the overlapping 0.3 kb region with the 1.9 kb N-terminal Cas9v2nickase fragment to reconstitute the full-length dCas9v2-VP64 (Herpes simplex virus

transcriptional activator domain) by HDR. The T2 sequence was added adjacent to each homology arm to cut out the donor DNA from the AAV vector genome. The vector sequence is shown in Supplementary Table S1. **B** shows the genome structure after integration of the donor by HDR. Immunoblot analyses of the blasticidin-resistant HEK-Cas9v1-Myc#2+dCas9v2(full)-VP64#1 and #2 cells were shown in Supplementary Fig S6. Primers (P5-P6) were set to detect the Bsd genes in the AAVS1 locus after HDR. *The puromycin resistant gene is not translated to the protein because of the stop codon following the blasticidin resistance gene.

Supplementary Fig S9
Activation of endogenous IFNβ gene promoter via reconstituted dCas9v2-VP64. To examine the function of reconstituted dCas9v2-VP64 in sgLenti #1 and #2 cells, in Supplementary S6, we examined endogenous IFN(interferon)β gene promoter activation via dCas9v2-VP64, MS2(bacteriophage coat protein)-P65(NF-κB trans-activating subunit)-HSF1(human heat shock factor 1), and MS2(aptamer)-sgRNA, targeting the IFNβ gene promoter site. To monitor the activation level of the IFNβ gene, a reporter ISRE (interferon-stimulated response element)- luciferase plasmid was co-transfected with a MS2-P65-HSF1 plasmid, and MS2-sgRNA targeting the IFNβ gene promoter site, or control sgRNA. **A.** Schematic representation of the study. **B.** Luciferase activity was significantly activated by MS2-sgRNA targeting IFNβ (12.8- and 19.0-fold to the control sgRNA in clones #1 and #2, respectively). *indicates a significant difference, compared with corresponding control sgRNA (p<0.01).

Supplementary Fig S10
Lentivirus constructs to express Myc-tagged Cas9 nickase termed Cas9(Nick)-Myc. A, B. Cas9(Nick)-Myc was constructed by replacing the N-terminal of Cas9v1 with Cas9v2 nickase at the BspHI restriction enzyme site; to differentiate reconstituted Cas9v2 integrated into the AAVS1 locus from the Cas9(Nick)-Myc randomly integrated into the host genome (the first step of Fig7). **C.** Following the lentivirus infection and hygromycin selection, Cas9(Nick)-Myc expression was confirmed using an anti-Cas9 antibody.

Supplementary Fig S11
Construction of AAV vector #7 to introduce the 1.9 kb N-terminal Cas9v2 nickase into the AAVS1 locus. A. AAV vector #7 was constructed to employ lentiviral Cas9(Nick)-Myc in the genome; to integrate the 1.9 kb N-terminal Cas9v2 nickase into the AAVS1 locus (the second step of Fig 7) via the addition of sgRNA7 (in red), and by replacing the T1 target sequence of AAV #5, with T7/1 (in green). **B.** There is a possible DNA sequence at the AAVS1 locus to make DSB with Cas9 nickase. The target sequence, T7/1, was designed based on the AAVS1 sequence, replacing the 14bp offset with 4bp offset for convenience.

Supplementary Fig S12
Construction of AAV vector #8 to reconstitute full-length Cas9v2-FLAG nickase in the AAVS1 locus. A. AAV vector #8 was constructed to employ lentiviral Cas9(Nick)-Myc for reconstitution of full-length Cas9v2-FLAG nickase using HDR (the third step of Fig 7); by adding sgRNA8 (in red) and replacing the T2 target sequence of AAV #2, with T8/2 (in green). **B.** There is a possible DNA sequence at the junction between the 1.9 kb N-terminal Cas9v2 and P2A, which may make a DSB in the DNA sequence with Cas9

nickase. The target sequence, T8/2, was designed based on the AAVS1 sequence, replacing the 8bp offset with a 4bp offset, for convenience. Lentiviral Cas9(Nick)-Myc remained intact as the sequence containing the linker and P2A, recognized by sgRNA2, was only present in the junction, and not in lentiviral Cas9(Nick)-Myc.

Supplementary Fig S13

Reconstitution of full-length dCas9-VP64 in the AAVS1 locus A. Schematic representation of AAV vector #9 and the AAVS1 locus around the target sequence, as in the third step of the strategy outlined in Fig 7; for full-length dCas9-VP64 reconstitution rather than full-length Cas9v2-FLAG nickase. **B**. Genomic structure following integration with HDR. PCR primers P1-P3 (Cas9v2), and P4-P2 (Puro), were used to examine whether full-length dCas9v2-VP64 was properly integrated into the AAVS1 locus at the 5′- and 3′- regions.

Supplementary Fig S14

Lentiviral genomic integration of Cas9(Nick)-Myc in all the clones. Lentiviral Cas9(Nick)-Myc proteins were not detected in clones #1, #2, or #3 of the reconstitute full-length Cas9v2nickase-FLAG cells (Fig 9D), and not in clones #1, #3, or #6 of the reconstitute dCas9v2-VP64 cells (Fig 10D). **A**. The 0.9 kb PCR products, using P7-P3 primers, indicated that all the six clones had the Cas9(Nick)-Myc genes in their genomes; similar to parent Cas9(Nick)-Myc#2 cells. **B**. Primers (P7-P3) were designed to detect lentiviral Cas9(Nick)-Myc in the genome.

Supplementary Fig S15

Comparison of HEK293T-Cas9v2nickaseNT+dCT-VP64 cell genomic structures (clones #1- #6). A, B. Although all six clones had reconstituted full-length dCas9v2-VP64 in the AAVS1 locus (Fig 10), clone #4 had a deletion around the right arm. **C**. As shown in Fig 10B, the PCR product of clone #4, using P4-P2 (Puro), was only 0.2 kb, which was much shorter than that of clones #1, #2, #3, #5, and #6 (1.1 kb). The deletion in clone #4 confirmed that its PCR product, using P5-P2 (Bsd), was 0.9 kb.

Supplementary Fig S16

HEK293T cells expressing lentiviral Cas9(Nick)-Myc without drug selection. A. HEK293T cells (500/well) were infected with 1,000 transduction units of lentivirus vector to express Cas9(Nick)-Myc (MOI=2), for the first step. Infected cells were plated in a 6-well plate with three different dilutions (1/30, 1/10, and 1/3). After ten days without drug selection, clearly isolated colonies (clones #1- #10) were analyzed with an anti-Cas9 antibody. **B**. The same experiment as in **A** was carried out independently.

Supplementary Fig S17

PCR and immunoblot to confirm the reconstitution of full-length Cas9v2nickase-FLAG without drug selection. HEK293T-Cas9v1-Myc+Cas9v2nickase-NT#9 cells (500/well) were seeded in a 24-well plate and infected with 10,000 transduction units of AAV vector #2 (MOI=20), for the third step. Infected cells were plated in a 6-well plate with three different dilutions (1/30, 1/10, and 1/3). Isolated colonies (clones #1–#6) were analyzed to determine full-length Cas9v2nickase-FLAG reconstitution efficiency. **A**. Genomic DNA prepared from the indicated cells were analyzed using PCR, with primers P5-P6. The 1.2 kb PCR products, indicating integration of the donor elements (Bsd gene), were detected

in all clones. Lysates obtained from the cells used for the PCR analysis were analyzed using immunoblotting to detect reconstituted full-length Cas9v2nickase-FLAG (**B**) and Cas9v1-Myc (**C**). **D**. β-actin served as the loading control.

Supplementary Fig S18

Removal of randomly integrated lentivirus containing Cas9v1-Myc from the genome without drug selection. Five hundred HEK293T cells expressing Cas9v1-Myc and full-length Cas9v2-FLAG (Cas9v1-Myc +Cas9v2-FLAG #7), in a 24-well plate, were infected with AAV vector #3 (MOI=20), for the fourth step. Infected cells were plated in a 6-well plate with three different dilutions (1/30, 1/10, and 1/3). After ten days without drug selection, clearly isolated colonies were collected from the 6-well plate, and their genomic DNA was analyzed using PCR, with P7-P8 primers (for Cas9v1) (**A**) and P1-P3 primers (for Cas9v2) (**B**). Lentiviral Cas9v1 was successfully removed from the genome in four out of ten. In addition, after 6% SDS-PAGE, lysates obtained from the cells used for the PCR analysis were analyzed using immunoblot, with antibodies to detect lentiviral Cas9v1-Myc (**C**), and the reconstituted full-length Cas9v2-FLAG (**D**). β-actin served as the loading control. (**E**). In this experiment, lentiviral Cas9v1 was removed from four of the ten colonies tested.

Supplementary Fig S19

The integration of modified Cas9s minimizes off-target effects at the AAVS1 locus. To reduce possible off-target effects, various modified Cas9s can be introduced in the AAVS1 locus, using non-integrating lentiviral Cas9(Nick)-Myc or AAV-SaCas9 (*Staphylococcus aureus,* Cas9 is small enough for AAV packaging), with minimum genetic risk in the first step, because they are not integrated in the host genome. **A-C** shows reconstitution of Cas9v2 nickase in the AAVS1 locus via the transient expression of Cas9(Nick)-Myc or SaCas9. (1) Co-infection of non-integrating lentivirus, to express Cas9(Nick)-Myc, and AAV vector (sgRNA1/7 + Donor (modified Cas9v2 N-terminal)). (2) During the transient expression of Cas9(Nick)-Myc, sequential infection of AAV (sgRNA2/8 + Donor (0.3 kb overlap + modified Cas9v2 C-terminal)) should be performed within a short interval (36 h). Schematic structures of modified Cas9s, the Cas9v2 nickase; dCas9-*Fok*I, deactivated Cas9 fused to *Fok*I catalytic endonuclease; eSpCas9(1.1), enhanced specificity *Streptcoccus pyogens* Cas9; SpCas9-HF, high fidelity SpCas9 with specific mutations are shown in brackets. These may be reconstituted in the AAVS1 locus using the same procedure as for Cas9v2 nickase.

Supplementary Table S1

AAV vector #1 sequence is provided as an Excel file (Table S1-AAV vector #1).
AAV vector #2 sequence is provided as an Excel file (Table S1-AAV vector #2).
AAV vector #5 sequence is provided as an Excel file (Table S1-AAV vector #5).
AAV vector #6 sequence is provided as an Excel file (Table S1-AAV vector #6).
AAV vector #7 sequence is provided as an Excel file (Table S1-AAV vector #7).
AAV vector #8 sequence is provided as an Excel file (Table S1-AAV vector #8).
AAV vector #9 sequence is provided as an Excel file (Table S1-AAV vector #9).

AAV vector #3 sequence as for a pair of sgRNAs.

Gene name	Sequence (5' to 3')
sgRNA3-1 (specific)	gGCCCGTCTGTTGTGTGACTC
sgRNA scaffold	GTTTTAGAGCTAGAAATAGCAAGTTAAAATAAGGCTAGTCCGTTATCAACTTGAAAAAGTG GCACCGAGTCGGTGCTTTTTTTGGtgtaca
sgRNA3-2 (specific)	gACTCAAGGCAAGCTTTATTG
sgRNA scaffold	GTTTTAGAGCTAGAAATAGCAAGTTAAAATAAGGCTAGTCCGTTATCAACTTGAAAAAGTG GCACCGAGTCGGTGCTTTTTTTGGtgtaca

AAV vector #4 sequence as for a pair of sgRNAs.

Gene name	Sequence (5' to 3')
sgRNA4-1 (specific)	gGACCCTAGTGCTCGTCGCCG
sgRNA scaffold	GTTTTAGAGCTAGAAATAGCAAGTTAAAATAAGGCTAGTCCGTTATCAACTTGAAAAAGTG GCACCGAGTCGGTGCTTTTTTTGGtgtaca
sgRNA4-2 (specific)	gCCAGGAGGACGACCATCATC
sgRNA scaffold	GTTTTAGAGCTAGAAATAGCAAGTTAAAATAAGGCTAGTCCGTTATCAACTTGAAAAAGTG GCACCGAGTCGGTGCTTTTTTTGGtgtaca

Supplementary Table S2

Genomic sequences are provided in Excel.

Cas9v2-NT1.9k clone #4: Table S2-Genome-NT.
Reconstituted full-length Cas9v2-FLAG #7: Table S2-Genome-Full.
Cas9v2nickase-NT1.9k clone #9: Table S2-Genome-NTnick
Reconstituted full-length Cas9v2 nickase-FLAG #3: Table S2-Genome-Fullnick
Reconstituted full-length dCas9v2-VP64 #1: Table S2-Genome-Fulld-VP64
.

Supplementary Table S3

Primer sequences for PCR.

Name	Sequence (5' to 3')
P1	CGGACCACTTTGAGCTCTACTGGC
P2	GAACACCTAGGACGCACCATTCTCAC
P3	AACAGCAGGGCTCCGATCAGGTTC
P4	AACCTCCCCTTCTACGAGCGGCTC
P5	TTGCTGCCCTCTGGTTATGTGTGGG
P6	CAGGAGACTAGGAAGGAGGAGGCCT
P7	CGTTACATAACTTACGGTAAATGG
P8	TTAGCATCCACTCCACTTGCGTTA
P9(IFNAR1)	TGTGTGTCAGAAGAGGCGGC
P10(IFNAR1)	GGCAAAGTGGACCGAAGGTT
P11(puromycin)	GAGTACAAGCCCACGGTGCGCCT
P12(puromycin)	GGAACCGCTCAACTCGGCCAT

Supplementary Table S4

MS2-sgRNA sequence.

Name	Sequence (5' to 3')
sg-IFNβ	gCTGAAAGGGAGAAGTGAAAG
sg-control	CCGTGACACGTCTC

Supplementary Fig S1

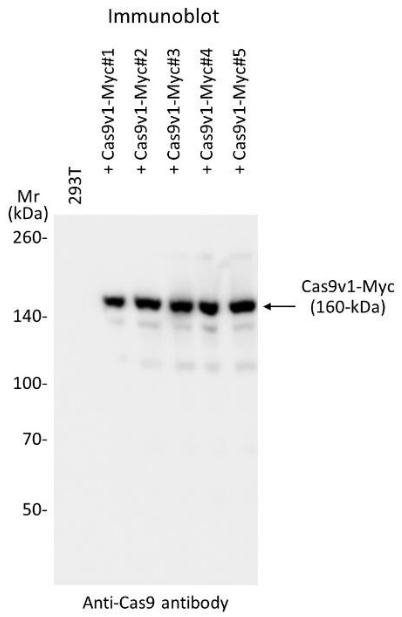

Supplementary Fig S2

A Allele exhibiting the 4.5kb PCR product (Cas9v2-NT#8 in Fig 3A)

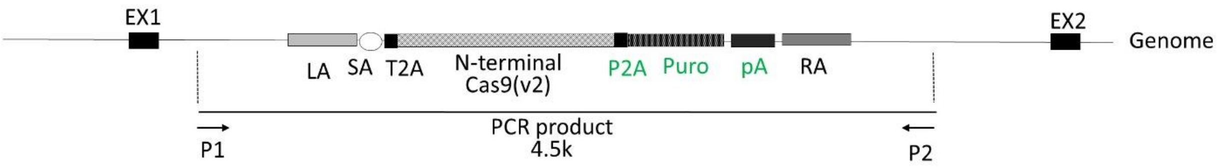

B Allele exhibiting the 4.2kb PCR product (Cas9v2-NT#8 in Fig 3A)

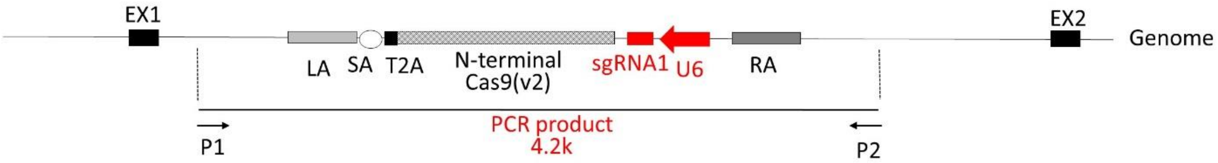

Supplementary Fig S3

A AAV vector #4

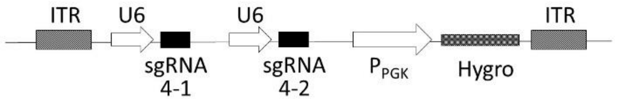

B HEK293T-Cas9v2-FLAG + sgLenti#10 Cells (in Fig 6)

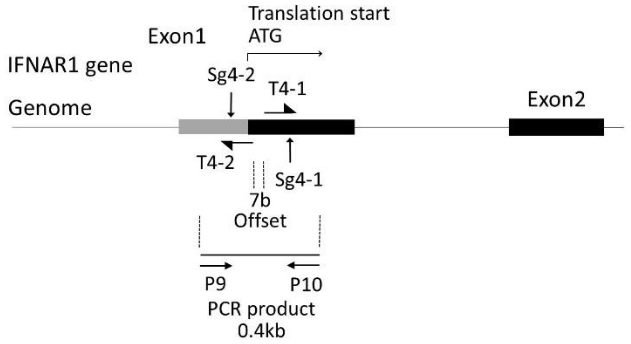

Supplementary Fig S4

DNA sequence analysis of independent two clones (#1 and #3) after infection of AAV#4 expressing sgIFNAR1 in Supplementary Fig S3

A

IFNAR1 gene (sgRNA4-1 → on upper strand; ← sgRNA4-2 on lower strand)

```
catgtaactggtgggatctgcggcggctcccagATGATGGTCGTCCTCCTGGGCGCGACGACCCTAGTGCTCGTCGCCGTGGCGCCATGGGTGTTG
                                 M  M  V  V  L  L  G  A  T  T  L  V  L  V  A  V  A  P  W  V  L
```

B sgIFNAR1 clone #1

```
catgtaactggtgggatctgcggcggctccca---------------tctgggcgcgacgaccctagtgctc----CCGTGGCGCCATGGGTGTTG

catgtaactggtgggatctgcggcggctcccagAT-----------------------------------------CCGTGGCGCCATGGGTGTTG
```

C sgIFNAR1 clone #3

```
catgtaactggtgggatctgcggcggctcccagATG---GTCGTCCTCCTGGGCGCGACGAC--------------CCGTGGCGCCATGGGTGTTG
                                 M     V  V  L  L  G  A  T  T                R  G  A  M  G  V

catgtaactggtgggatctgcggcggctcccagAT-----------------------------------------CCGTGGCGCCATGGGTGTTG
```

Supplementary Fig S5

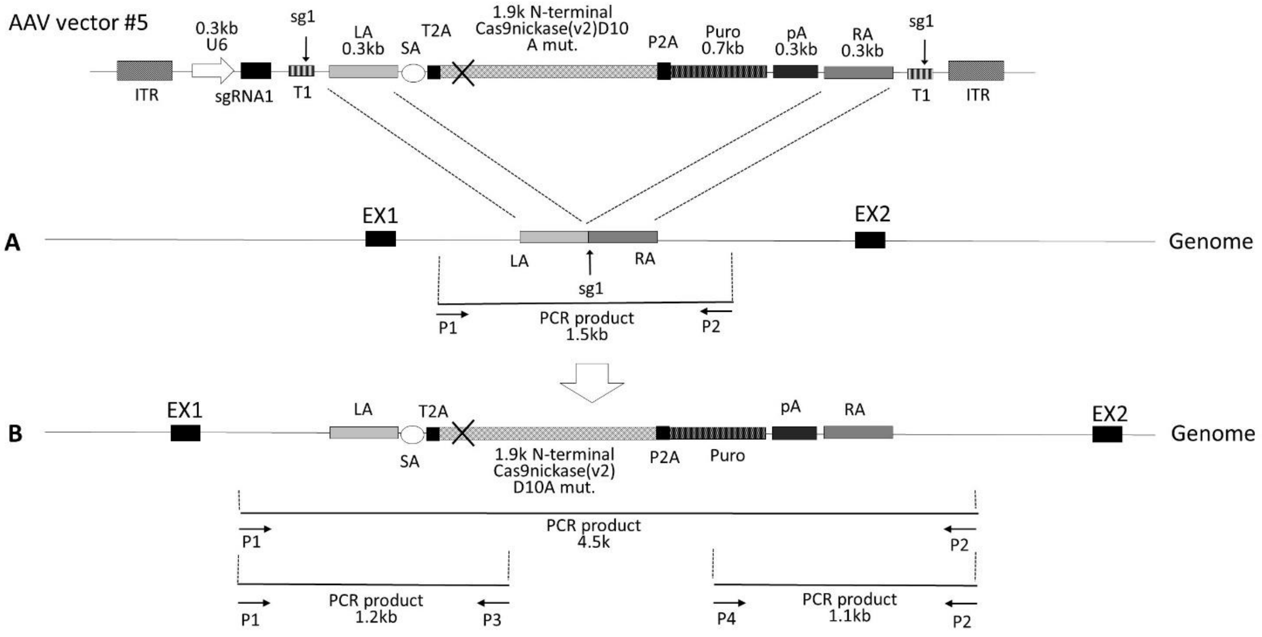

HEK293T-Cas9v1-Myc#2+Cas9v2nickase-NT#7 and #9 cells in Supplementary Fig S6

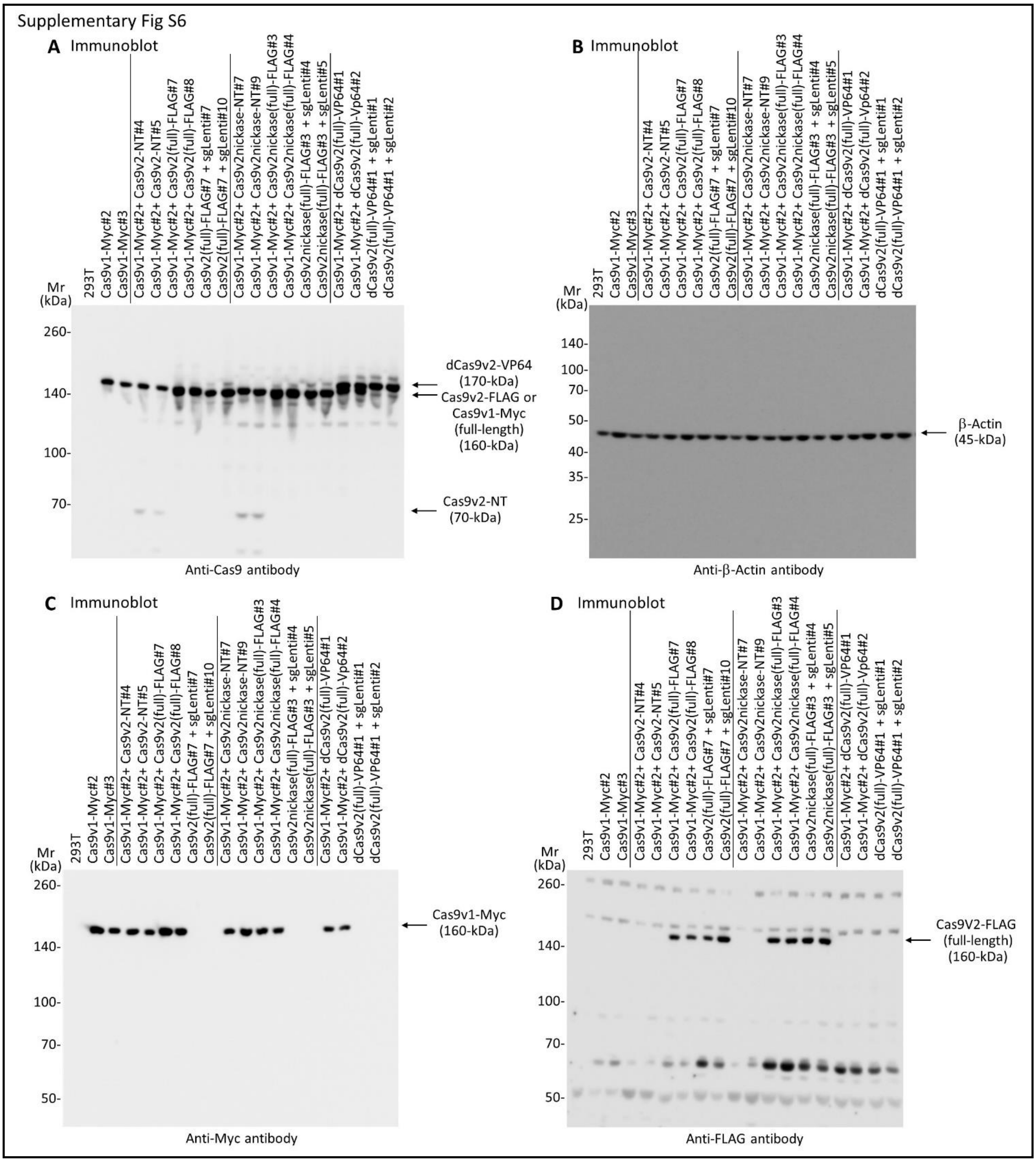
Supplementary Fig S6
A Immunoblot
293T
Cas9v1-Myc#2
Cas9v1-Myc#3
Cas9v1-Myc#2+ Cas9v2-NT#4
Cas9v1-Myc#2+ Cas9v2-NT#5
Cas9v1-Myc#2+ Cas9v2(full)-FLAG#7
Cas9v1-Myc#2+ Cas9v2(full)-FLAG#8
Cas9v2(full)-FLAG#7 + sgLenti#7
Cas9v2(full)-FLAG#7 + sgLenti#10
Cas9v1-Myc#2+ Cas9v2nickase-NT#7
Cas9v1-Myc#2+ Cas9v2nickase-NT#9
Cas9v1-Myc#2+ Cas9v2nickase(full)-FLAG#3
Cas9v1-Myc#2+ Cas9v2nickase(full)-FLAG#4
Cas9v2nickase(full)-FLAG#3 + sgLenti#4
Cas9v2nickase(full)-FLAG#3 + sgLenti#5
Cas9v1-Myc#2+ dCas9v2(full)-VP64#1
Cas9v1-Myc#2+ dCas9v2(full)-Vp64#2
dCas9v2(full)-VP64#1 + sgLenti#1
dCas9v2(full)-VP64#1 + sgLenti#2
Mr (kDa)
260-
140-
100-
70-
dCas9v2-VP64 (170-kDa)
Cas9v2-FLAG or Cas9v1-Myc (full-length) (160-kDa)
Cas9v2-NT (70-kDa)
Anti-Cas9 antibody
B Immunoblot
293T
Cas9v1-Myc#2
Cas9v1-Myc#3
Cas9v1-Myc#2+ Cas9v2-NT#4
Cas9v1-Myc#2+ Cas9v2-NT#5
Cas9v1-Myc#2+ Cas9v2(full)-FLAG#7
Cas9v1-Myc#2+ Cas9v2(full)-FLAG#8
Cas9v2(full)-FLAG#7 + sgLenti#7
Cas9v2(full)-FLAG#7 + sgLenti#10
Cas9v1-Myc#2+ Cas9v2nickase-NT#7
Cas9v1-Myc#2+ Cas9v2nickase-NT#9
Cas9v1-Myc#2+ Cas9v2nickase(full)-FLAG#3
Cas9v1-Myc#2+ Cas9v2nickase(full)-FLAG#4
Cas9v2nickase(full)-FLAG#3 + sgLenti#4
Cas9v2nickase(full)-FLAG#3 + sgLenti#5
Cas9v1-Myc#2+ dCas9v2(full)-VP64#1
Cas9v1-Myc#2+ dCas9v2(full)-Vp64#2
dCas9v2(full)-VP64#1 + sgLenti#1
dCas9v2(full)-VP64#1 + sgLenti#2
Mr (kDa)
140-
100-
70-
50-
40-
35-
25-
β-Actin (45-kDa)
Anti-β-Actin antibody
C Immunoblot
293T
Cas9v1-Myc#2
Cas9v1-Myc#3
Cas9v1-Myc#2+ Cas9v2-NT#4
Cas9v1-Myc#2+ Cas9v2-NT#5
Cas9v1-Myc#2+ Cas9v2(full)-FLAG#7
Cas9v1-Myc#2+ Cas9v2(full)-FLAG#8
Cas9v2(full)-FLAG#7 + sgLenti#7
Cas9v2(full)-FLAG#7 + sgLenti#10
Cas9v1-Myc#2+ Cas9v2nickase-NT#7
Cas9v1-Myc#2+ Cas9v2nickase-NT#9
Cas9v1-Myc#2+ Cas9v2nickase(full)-FLAG#3
Cas9v1-Myc#2+ Cas9v2nickase(full)-FLAG#4
Cas9v2nickase(full)-FLAG#3 + sgLenti#4
Cas9v2nickase(full)-FLAG#3 + sgLenti#5
Cas9v1-Myc#2+ dCas9v2(full)-VP64#1
Cas9v1-Myc#2+ dCas9v2(full)-Vp64#2
dCas9v2(full)-VP64#1 + sgLenti#1
dCas9v2(full)-VP64#1 + sgLenti#2
Mr (kDa)
260-
140-
100-
70-
50-
Cas9v1-Myc (160-kDa)
Anti-Myc antibody
D Immunoblot
293T
Cas9v1-Myc#2
Cas9v1-Myc#3
Cas9v1-Myc#2+ Cas9v2-NT#4
Cas9v1-Myc#2+ Cas9v2-NT#5
Cas9v1-Myc#2+ Cas9v2(full)-FLAG#7
Cas9v1-Myc#2+ Cas9v2(full)-FLAG#8
Cas9v2(full)-FLAG#7 + sgLenti#7
Cas9v2(full)-FLAG#7 + sgLenti#10
Cas9v1-Myc#2+ Cas9v2nickase-NT#7
Cas9v1-Myc#2+ Cas9v2nickase-NT#9
Cas9v1-Myc#2+ Cas9v2nickase(full)-FLAG#3
Cas9v1-Myc#2+ Cas9v2nickase(full)-FLAG#4
Cas9v2nickase(full)-FLAG#3 + sgLenti#4
Cas9v2nickase(full)-FLAG#3 + sgLenti#5
Cas9v1-Myc#2+ dCas9v2(full)-VP64#1
Cas9v1-Myc#2+ dCas9v2(full)-Vp64#2
dCas9v2(full)-VP64#1 + sgLenti#1
dCas9v2(full)-VP64#1 + sgLenti#2
Mr (kDa)
260-
140-
100-
70-
50-
Cas9V2-FLAG (full-length) (160-kDa)
Anti-FLAG antibody

Supplementary Fig S7

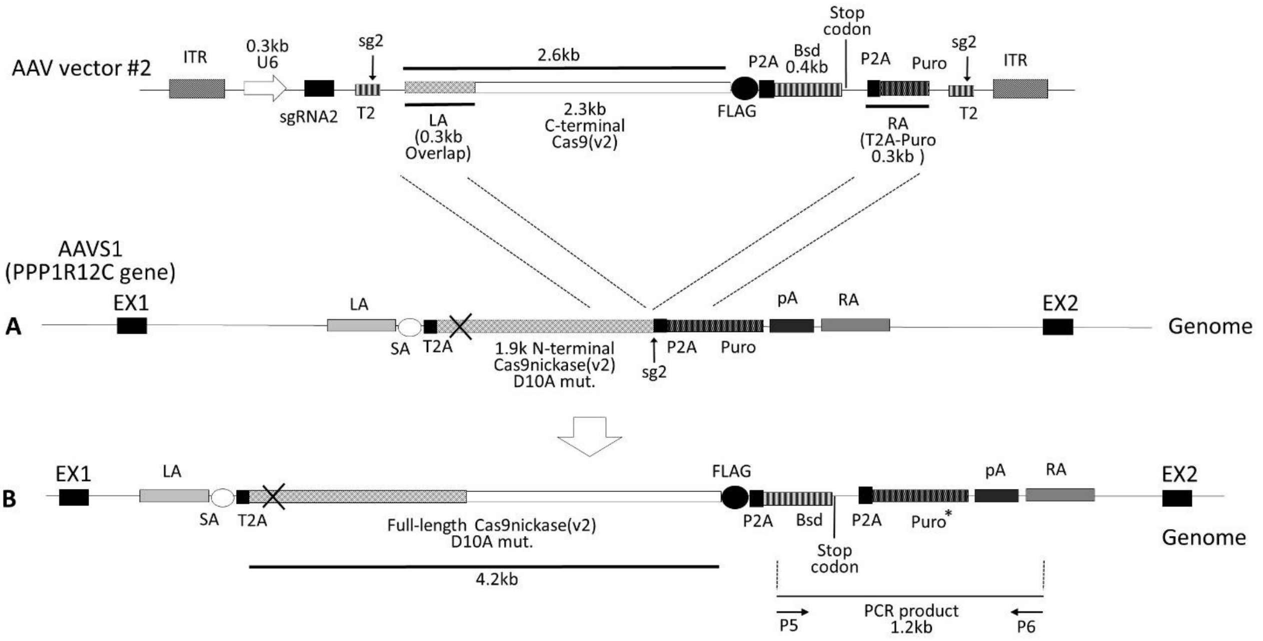

HEK293T-Cas9v1-Myc#2+Cas9v2nickase(full)-FLAG#3 and #4 cells in Supplementary Fig S6

Supplementary Fig S8

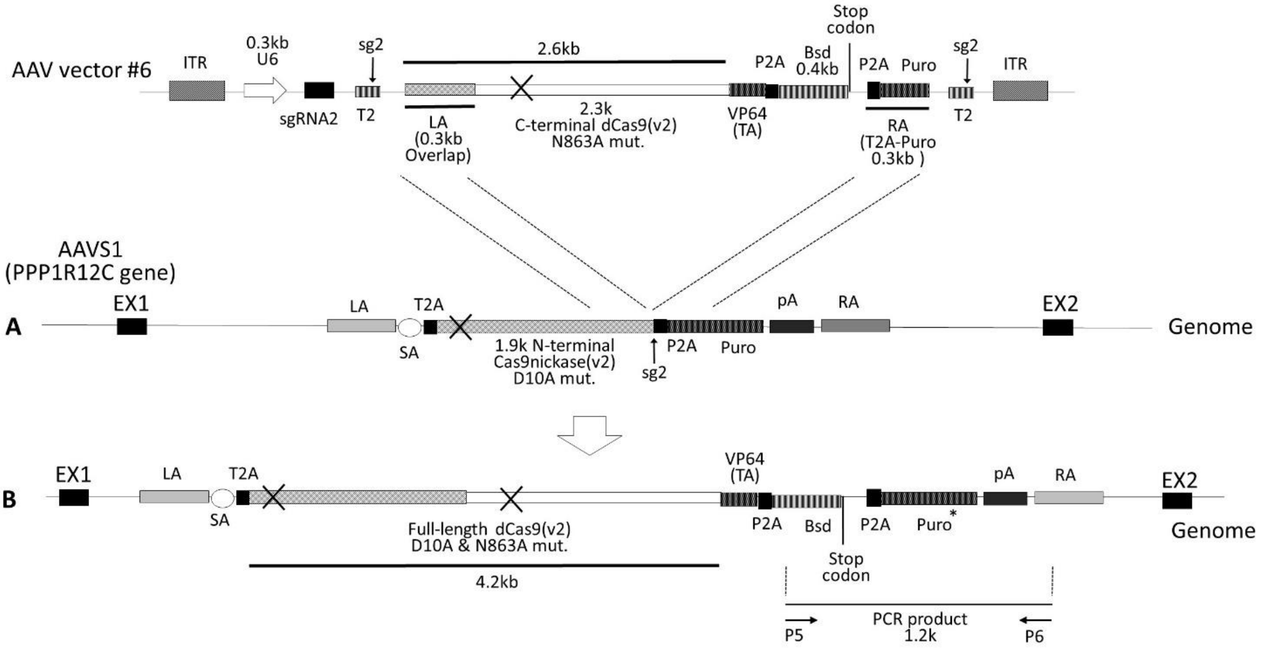

HEK293T-Cas9v1-Myc#2+dCas9v2(full)-VP64#1 and #2 cells in Supplementary Fig S6

Supplementary Fig S9 Activation of endogenous IFNβ gene in the cells expressing dCas9v2-VP64 with MS2-P65-HSF1 and MS2-sgIFNβ

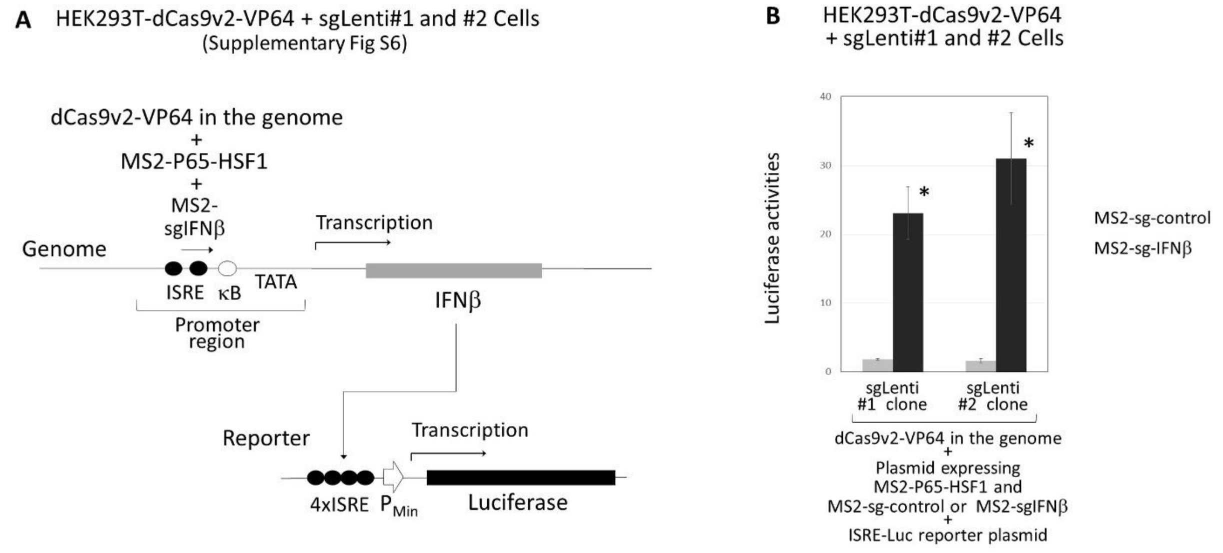

Supplementary Fig S10

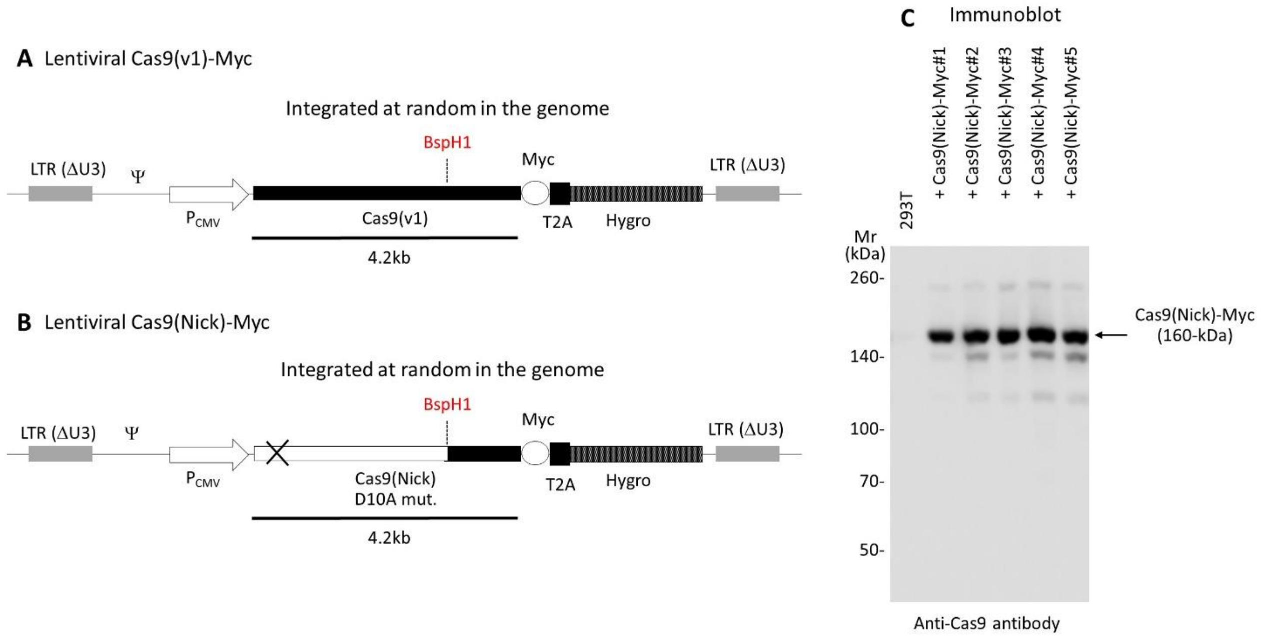

Supplementary Fig S11

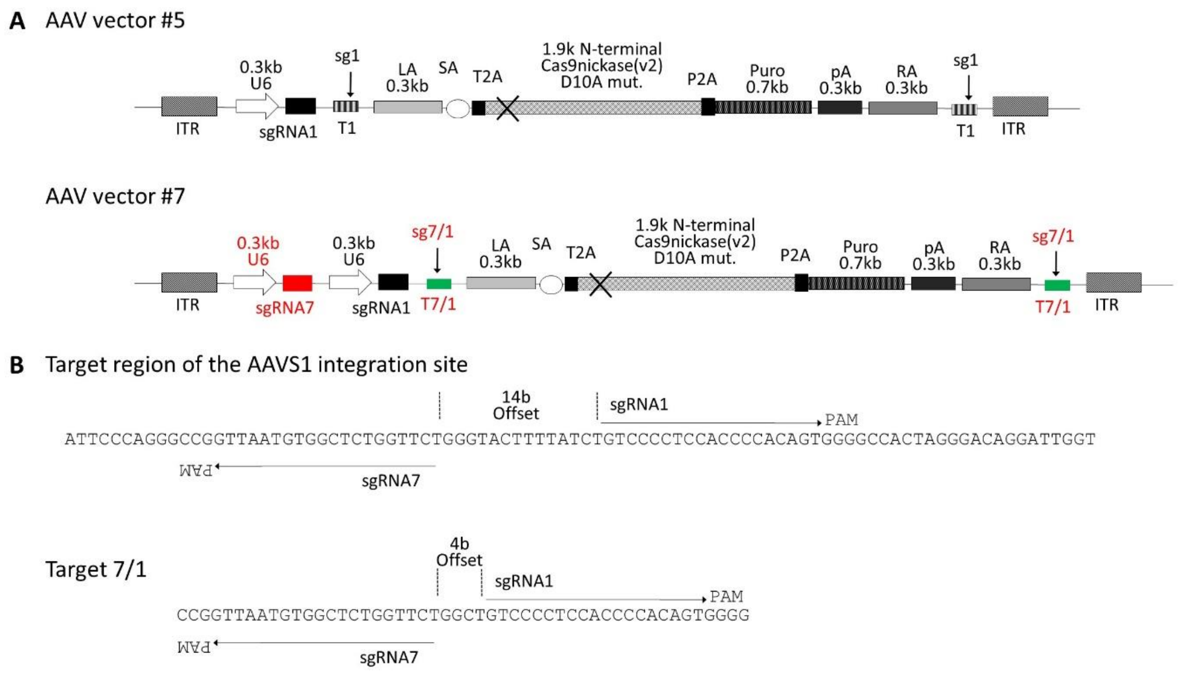

Supplementary Fig S12

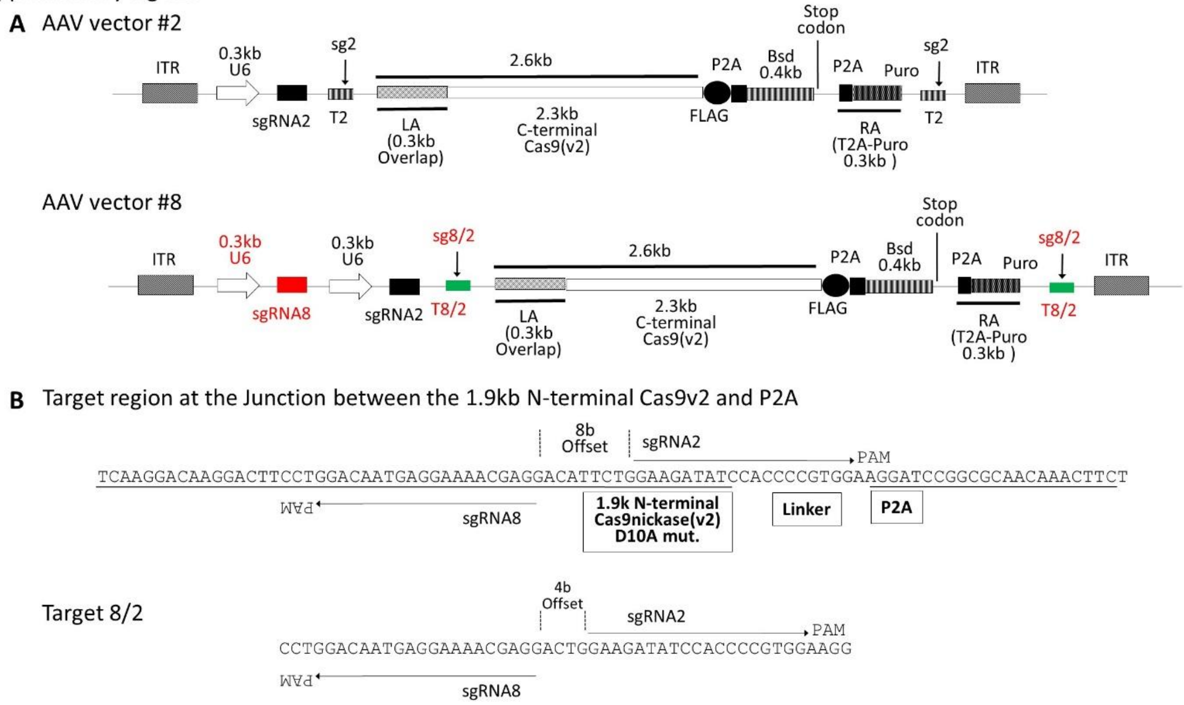

Supplementary Fig S13

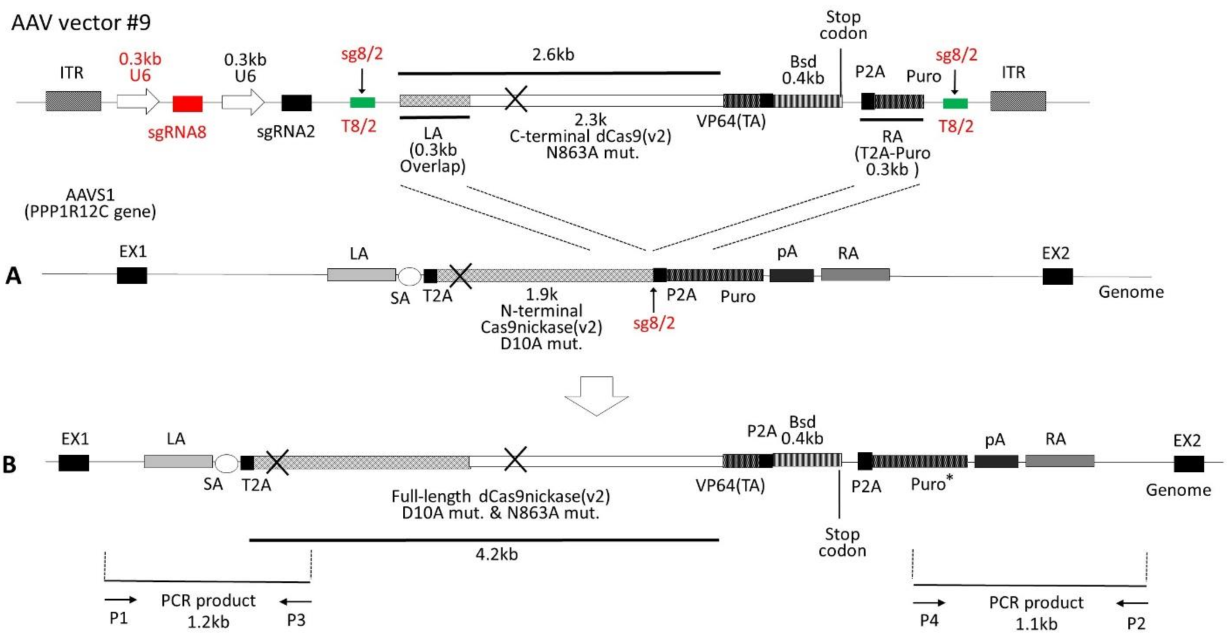

Supplementary Fig S14

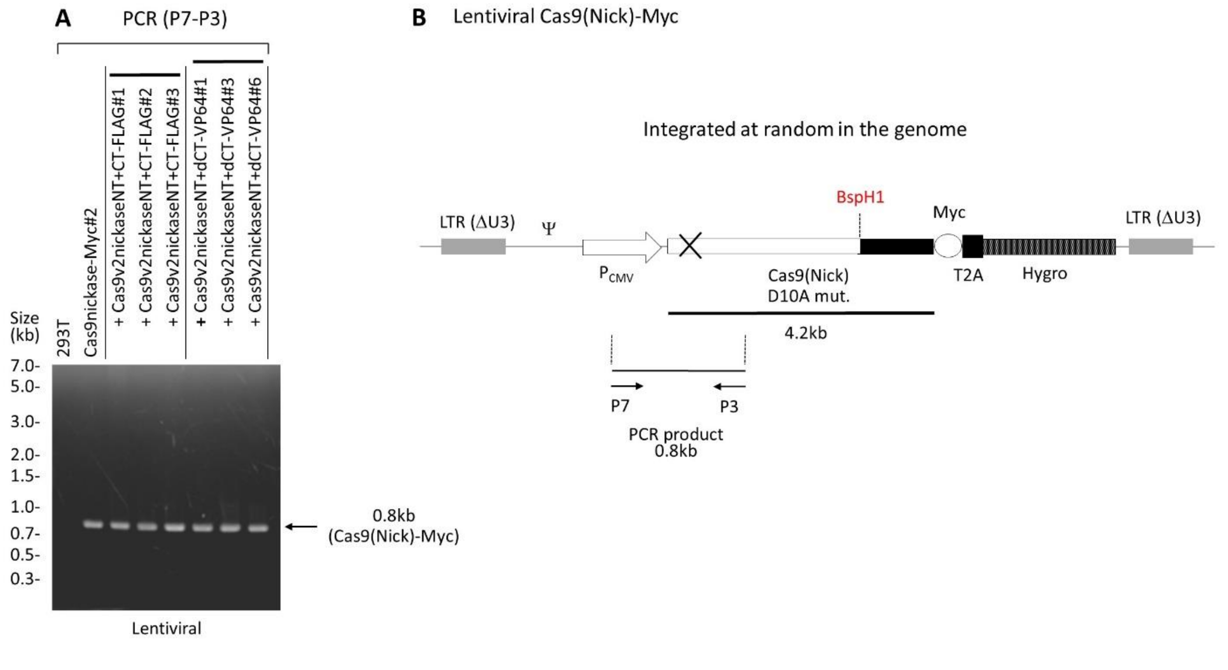

Supplementary Fig S15

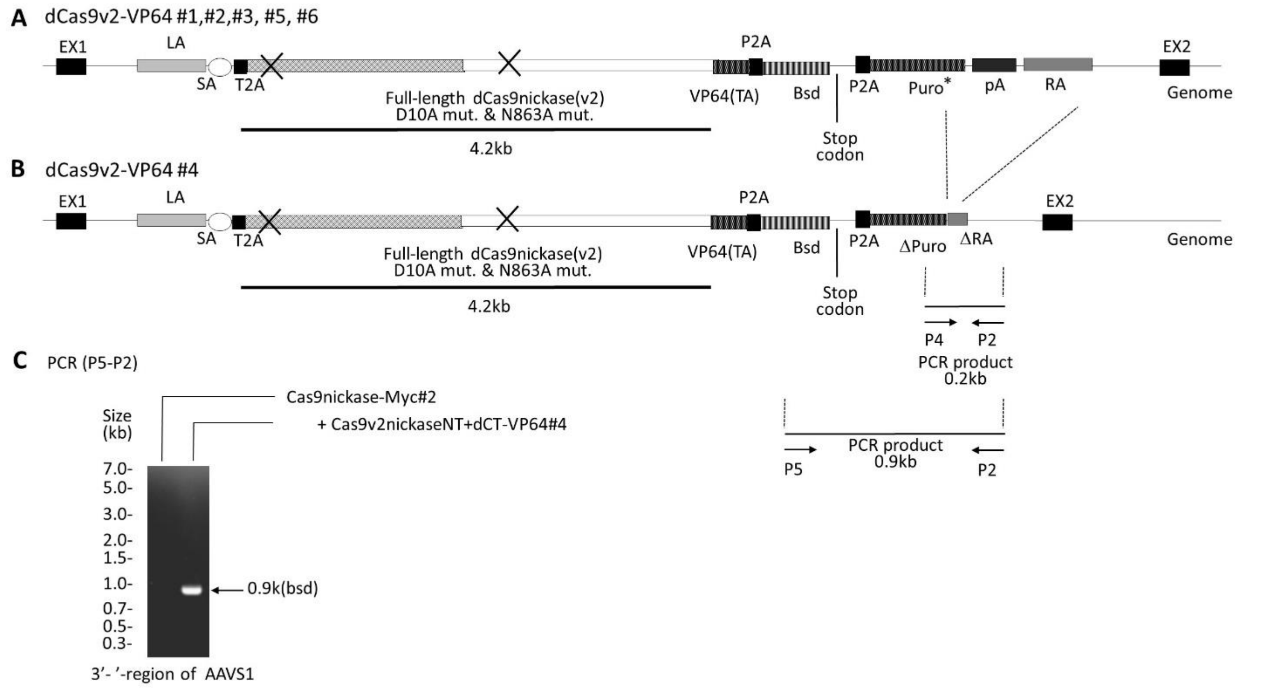

Supplementary Fig S16 Infection of Lentiviral Cas9(Nick)-Myc (**the first step**)

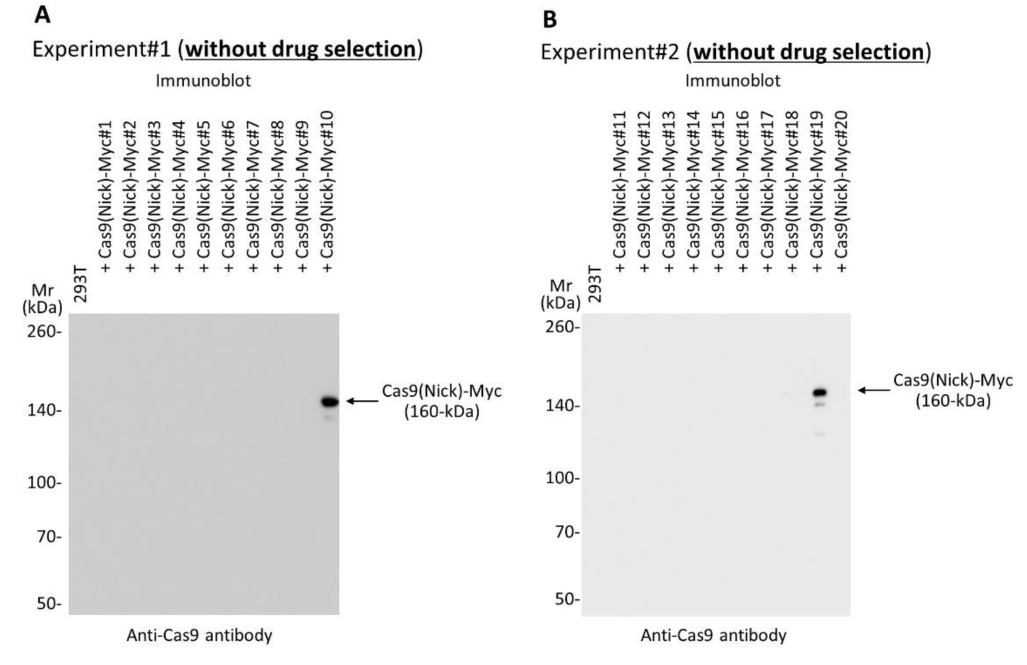

Supplementary Fig S17 Reconstitution of Cas9v2nickase(full)-FLAG with AAV#2 (**the third step**)

Experiment (**without drug selection**)

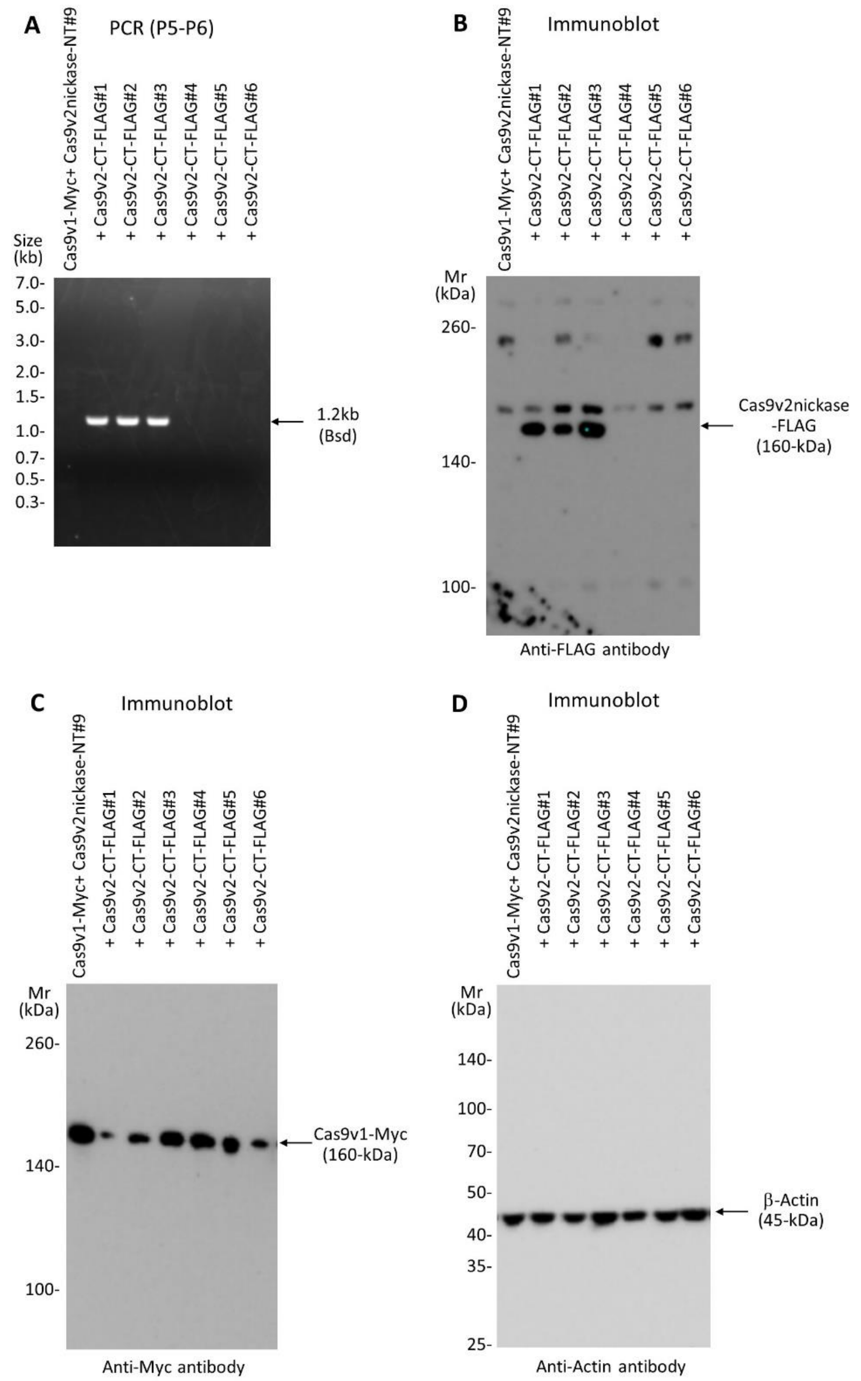

Supplementary Fig S18 Removal lentiviral Cas9v1-Myc from the Cas9v2(full)-FLAG-expressing cells with AAV#3 (**the fourth step**)

Experiment #3 (**without drug selection**)

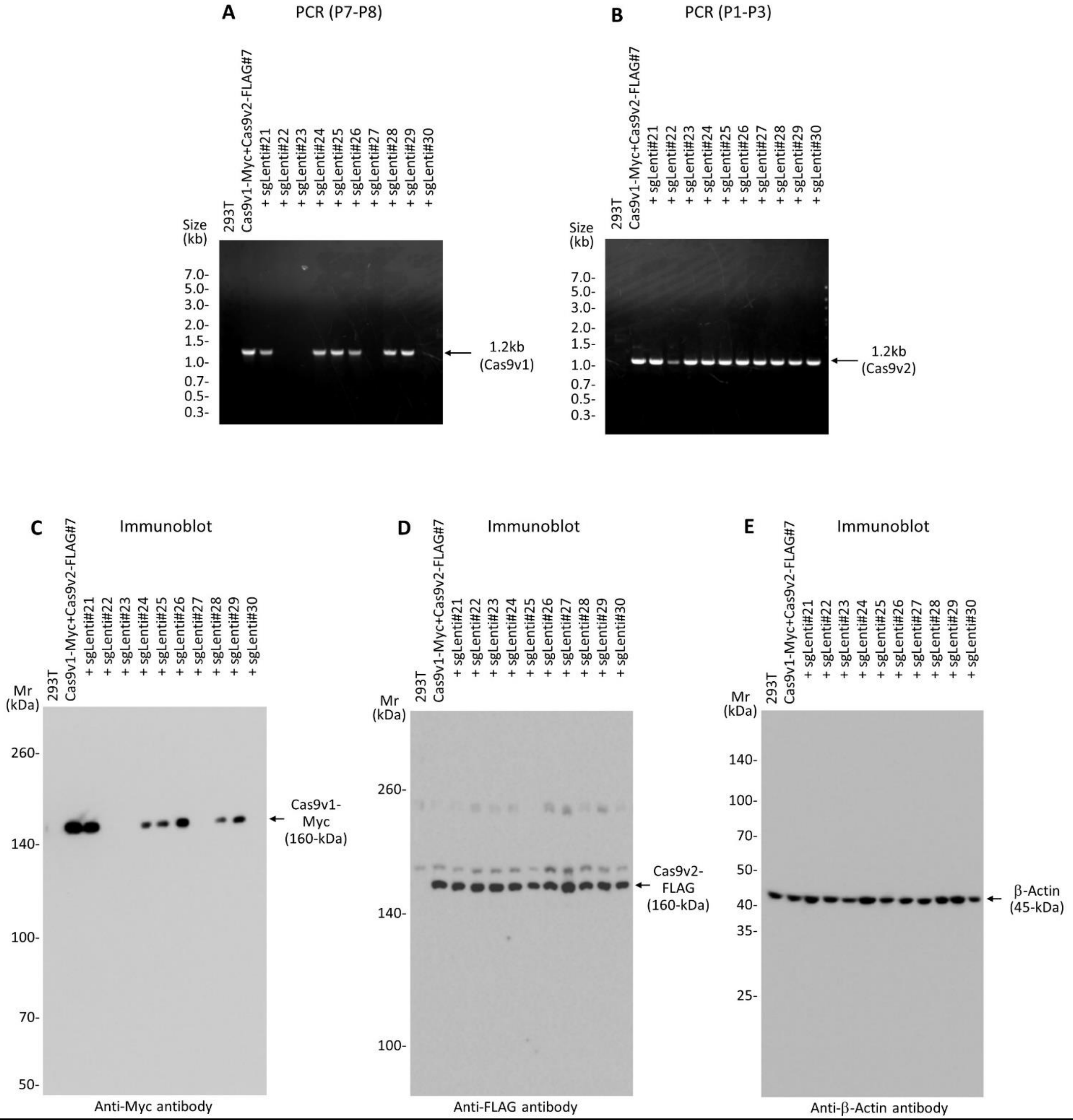

Supplementary Fig S19

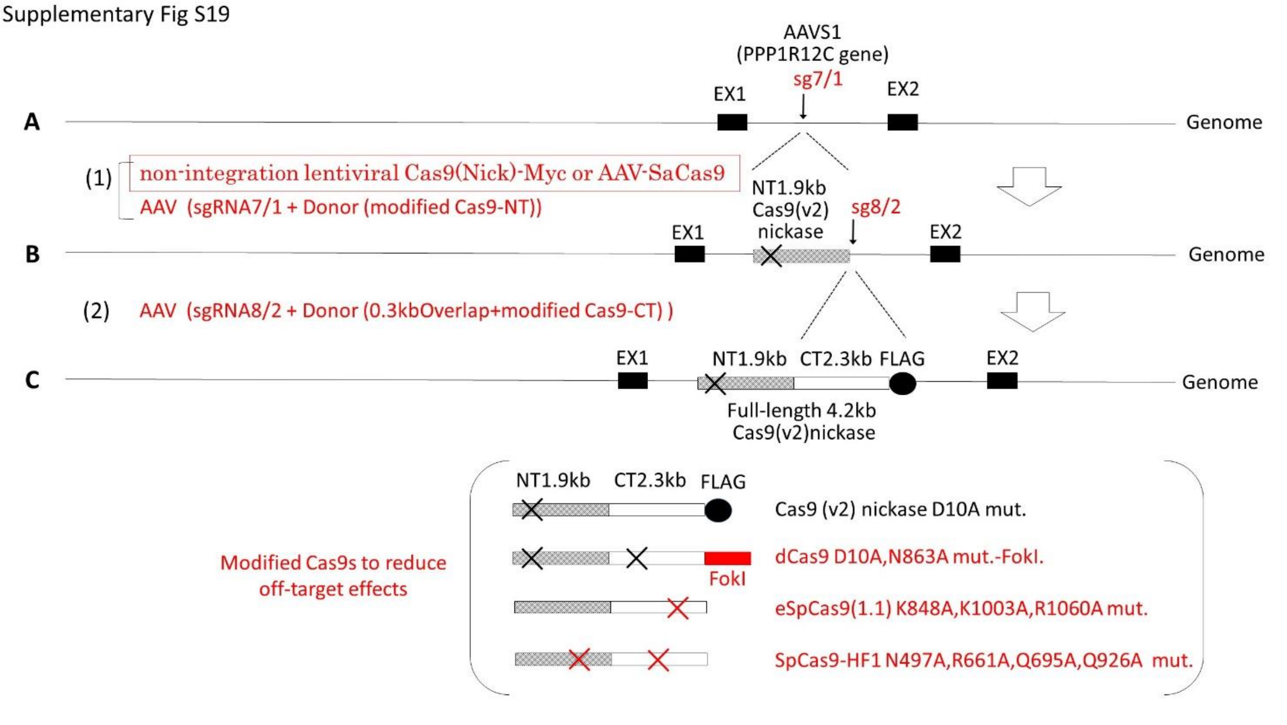

Supplement: Supplementary file 1 — Supplementary Information. [file 41598_2020_78450_MOESM1_ESM.pdf]
